# Autonomous Assessment of Medical Consent Forms: Development and Preliminary Feasibility Demonstration of the Universal Health Communication Index (UHCI)

**DOI:** 10.3390/healthcare14182956

**Published:** 2026-09-10

**Authors:** Cihat Özgüncü

**Affiliations:** Department of Neurology, Faculty of Medicine, Selcuk University, Akademi Mahallesi, Yeni İstanbul Caddesi No: 313, Selcuklu, 42130 Konya, Türkiye; cihatozguncu@gmail.com; Tel.: +90-332-241-5000

**Keywords:** informed consent, large language models, patient-centered care, health technology assessment, health policy, neurology

## Abstract

**Objective**: Informed consent documents often prioritize institutional liability over patient comprehension, and conventional readability metrics are inadequate to assess their ethical and cognitive complexities. This study aims to develop and demonstrate the preliminary feasibility of the Universal Health Communication Index (UHCI), an AI-driven Health Technology Assessment (HTA) framework utilizing large language models (LLMs) to objectively evaluate the multidimensional quality and operational impact of medical consent forms. **Methods**: The UHCI employs a hybrid natural language processing architecture to evaluate clinical texts across five dimensions: Accessibility, Transparency, Autonomy, System Burden, and Medical Adequacy. The pipeline deploys an ‘AI Persona’ to simulate patient cognitive load and an ‘AI Gold Standard’ utilizing clinical guidelines (UpToDate) to identify omitted risks. The framework was benchmarked in an exploratory proof-of-concept against a fifty-eight-member multidisciplinary human baseline and subsequently tested in a cross-lingual proof-of-concept by comparing standardized lumbar puncture consent forms across three distinct healthcare jurisdictions (Turkey, the UK, and the USA). **Results**: The algorithm effectively quantified abstract bioethical concepts into an objective HTA metric, demonstrating preliminary numerical agreement with the human baseline across five pilot forms (mean difference: 2.73, SD: 5.94). The framework effectively penalized the ‘illusion of transparency’ and defensive medical terminology. In the cross-lingual case study, although the global scores were comparable, the algorithm successfully discerned between forms prioritizing patient-centered autonomy and those reflecting directive institutional discourse. **Conclusions**: Transcending conventional readability formulas, the UHCI provides healthcare administrators and policymakers with a scalable, practical methodology to optimize clinical documentation. By identifying institutional bias and communicational barriers, this AI-assisted screening tool promotes equitable patient engagement and supports evidence-based health policies, demonstrating the responsible integration of AI into clinical workflows without replacing human clinical judgment.

## 1. Introduction

Informed consent documents are essential tools designed to safeguard patient autonomy and support the broader process of Shared Decision-Making (SDM) within provider–patient interactions [1]. However, while a well-designed document facilitates communication, it must be distinguished from the broader clinical process itself. Although this foundational bioethical imperative is embedded in international frameworks, including the Declaration of Helsinki and the Common Rule (45 CFR 46.116) [2,3,4], contemporary clinical workflows often reduce these documents to defensive administrative instruments that emphasize institutional liability over patient comprehension. Such forms employ complex legal language and directive institutional rhetoric, creating a significant cognitive burden for patients and systematically restricting their autonomy [5]. As a result, patients frequently sign these documents without a comprehensive understanding of the associated risks, perpetuating a structural information asymmetry that not only contravenes bioethical standards but also generates unintended organizational consequences and health economic inefficiencies that contradict evidence-based health policies [6].

In recent years, Natural Language Processing (NLP) and Large Language Models (LLMs) have transformed the summarization, analysis, and classification of clinical texts [7]. Nevertheless, most AI applications in the literature have primarily targeted electronic health records, radiology reports, or diagnostic coding [8,9]. As recent studies underscore, clinical risk communication remains especially prone to implicit misunderstandings, and current natural language models frequently fail to account for such pragmatic subtleties [10]. Moreover, a growing consensus emphasizes the need to progress beyond isolated algorithms and rigorously utilize ‘Agentic AI’ and human-in-the-loop (HITL) systems as systematic methodological tools to support formal Health Technology Assessment (HTA) and clinical governance [11]. Notably, while these forms are not the foundational elements of the consent process, they are critical communicative tools. Yet, there remains a need for multidimensional indices that can objectively evaluate their ethical, legal, linguistic, and medical sufficiency—which are key dimensions of patient-centered care and healthcare delivery. Conventional readability formulas [12] lack the capacity to assess semantic depth, transparency, or the anxiety induced in patients. This research gap underscores the necessity of an auxiliary, multidimensional evaluation methodology [13].

This study presents the Universal Health Communication Index (UHCI) algorithm, a computational framework designed to support the evaluation of clinical consent forms using established health policy and communication standards. The UHCI integrates XLM-RoBERTa [14]-based Zero-Shot classification—recently validated for pragmatic analysis in healthcare dialogues [10]—with Sentence-Transformer [15] semantic similarity (cosine similarity) and GPT-2-based perplexity metrics. This orchestration of models embodies recent progress in multi-agent clinical text extraction to provide a transparent and evidence-based evaluation process [16], while also incorporating conventional readability indices. To assess both clinical applicability and computational feasibility, test results are presented for standard neurology procedures, with a primary focus on lumbar puncture administration. By focusing on the structural and linguistic evaluation of clinical documentation, this methodology asserts that AI-assisted consent form screening can identify textual, informational, and communicational issues that may occasionally obstruct SDM. Ultimately, the UHCI is proposed as a supportive AI methodology to help decision-makers and healthcare professionals optimize clinical documentation, facilitating better patient engagement and the ethical integration of AI into clinical workflows.

## 2. Materials and Methods

### 2.1. Study Design and Data Sets

The study was structured in two phases. Phase 1 (Preliminary Feasibility and Proof-of-Concept) evaluated the algorithm’s descriptive alignment with human clinical baselines across a pilot set of five clinical forms to demonstrate its technical viability as an automated screening tool. Five standardized Turkish neurological consent forms [Tissue Plasminogen Activator therapy (tPA), plasma exchange (PE), botulinum toxin application (BTA), electromyography (EMG), and lumbar puncture (LP)] were acquired from the Turkish Neurological Association’s repository. Phase 2 (Cross-Lingual Proof-of-Concept) demonstrated the UHCI framework’s applicability by applying methodological control: comparing the exact same clinical procedure across different healthcare systems. The Turkish lumbar puncture (LP) form was juxtaposed with two English-language LP forms sourced from the USA (Austin Neuromuscular Center) and the UK. To accurately reflect standard UK clinical practice, the UK document was constructed by pairing the procedure-specific information leaflet from University Hospitals Plymouth NHS Trust with the nationally standardized NHS consent signature template.

### 2.2. Algorithm Development and Computational Environment

**Figure 1:**
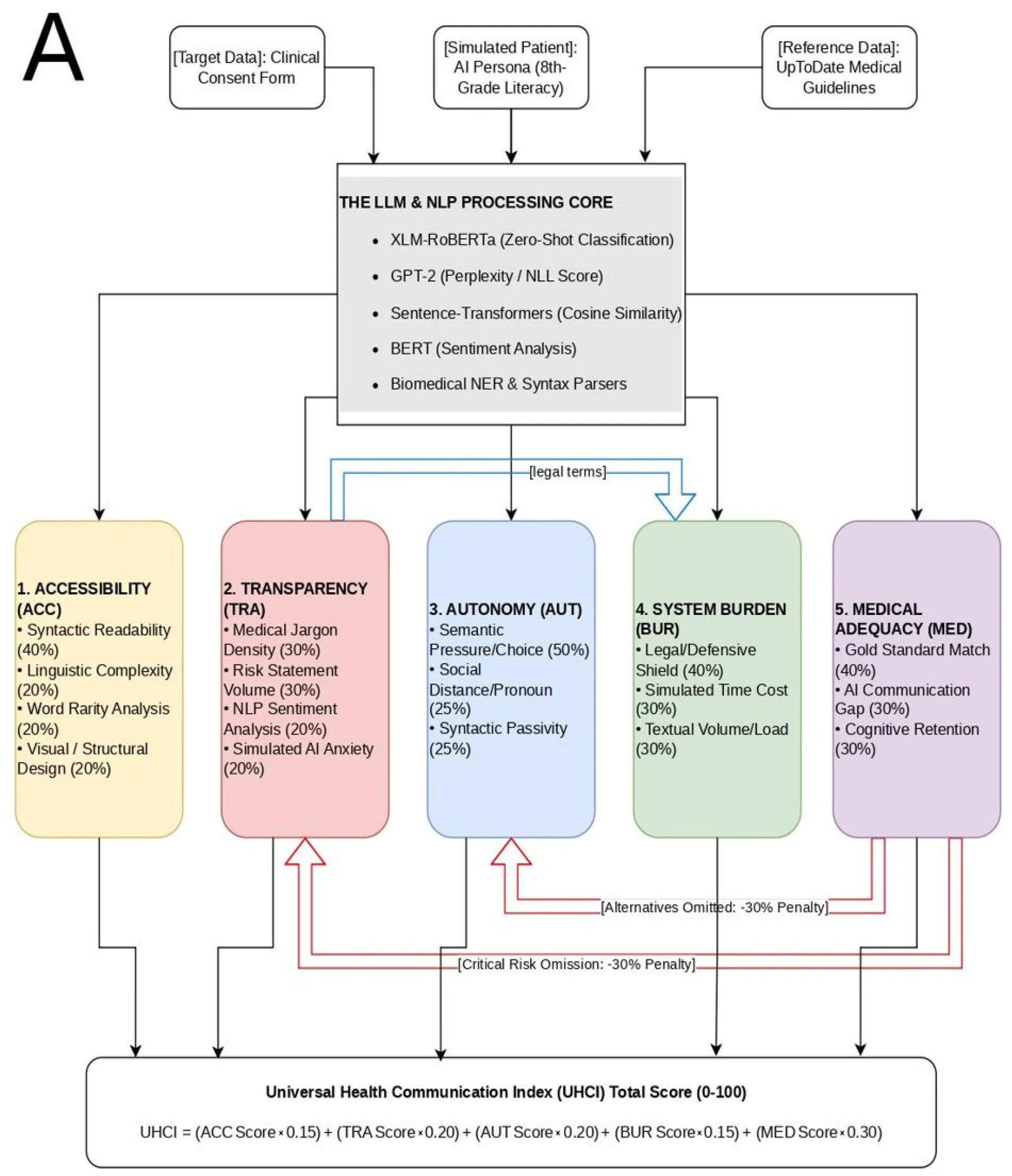

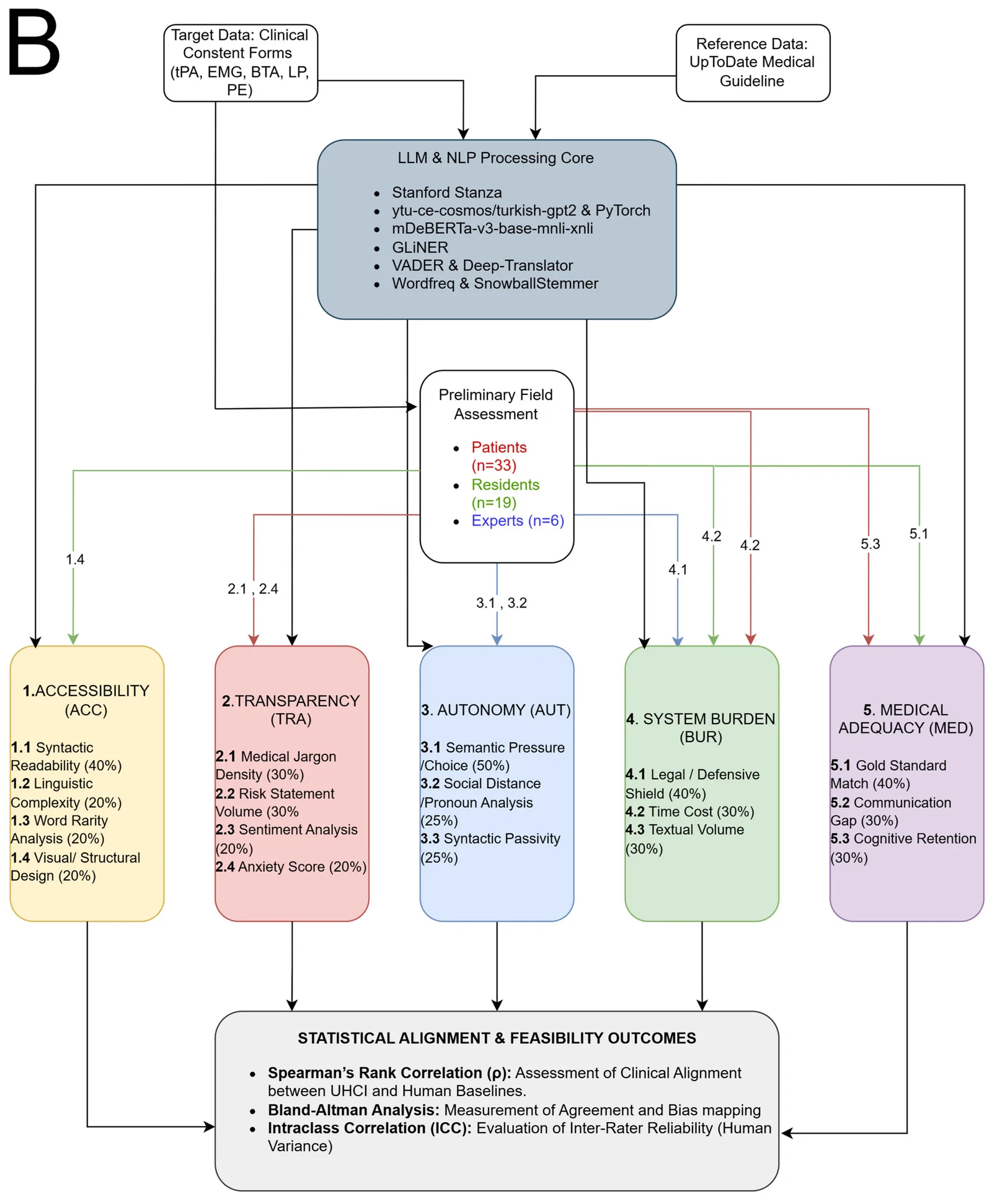
Computational Architecture and Multidisciplinary Assessment Framework of the Universal Health Communication Index (UHCI). (**A**) Computational Architecture and Data Processing Pipeline: The pipeline assesses clinical consent forms by comparing them to a reference gold standard (UpToDate) and a simulated AI patient persona. The core engine employs a hybrid ensemble of Large Language Models (LLMs) and Natural Language Processing (NLP) parsers to calculate 17 quantitative sub-parameters distributed across five primary dimensions: Accessibility (ACC), Transparency (TRA), Autonomy (AUT), System Burden (BUR), and Medical Adequacy (MED). Red dashed lines indicate autonomous cross-penalty mechanisms, in which critical risk omissions or the lack of clinical alternatives mathematically reduce transparency and autonomy scores, thereby preventing artificial score inflation and ensuring algorithmic accountability. (**B**) Multidisciplinary Assessment Workflow: The central node depicts the preliminary field assessment, demonstrating how the subjective evaluations of patients, residents, and clinical experts map directly onto their corresponding algorithmic sub-parameters. The color-coded arrows (red for laypersons, green for residents, blue for experts) illustrate the distributed assessment model, mapping each specific human sub-cohort strictly to the relevant UHCI modules they evaluated. The final stage outlines the statistical methodologies (Spearman’s ρ, Bland–Altman, and ICC) employed to measure the structural alignment and evaluate inter-rater variance between the autonomous outputs and the multidisciplinary human baseline. Abbreviations: ACC: Accessibility, AUT: Autonomy, BTA: Botulinum Toxin Application, BUR: System Burden, EMG: Electromyography, ICC: Intraclass Correlation Coefficient, LLM: Large Language Model, LP: Lumbar Puncture, MED: Medical Adequacy, NER: Named Entity Recognition, NLP: Natural Language Processing, PE: Plasma Exchange, tPA: Tissue Plasminogen Activator, TRA: Transparency, UHCI: Universal Health Communication Index.

The UHCI architecture was conceptualized using Google Gemini 3.1 Pro (Google LLC, Mountain View, CA, USA). Implementation and quantitative analyses were executed in Python (version 3.10, Python Software Foundation, Wilmington, DE, USA) via Google Antigravity and Google Colaboratory (Google LLC, Mountain View, CA, USA). For independent cross-verification purposes in the subsequent phases, the Anthropic Claude Sonnet 4.6 model (Anthropic PBC, San Francisco, CA, USA) was utilized. A detailed schematic of the computational architecture and the multi-agent workflow is presented in Figure 1A.

### 2.3. Simulated Data Flow: AI Persona and Gold Standard

To simulate patient cognitive load and comprehension, an ‘AI Persona’ was deployed through structured prompt engineering. The LLM was instructed to adopt the persona of an average patient with an 8th-grade health literacy level. The model evaluated the visual and structural design using the Patient Education Materials Assessment Tool (PEMAT) criteria [13,17], listed unfamiliar terms, identified questions, reported a Textual Alarm Index (0–10), and summarized three key recall points. To mitigate AI hallucinations, evaluations were repeated across three independent, memory-reset sessions [17].

To evaluate the medical adequacy of the forms, an ‘AI Gold Standard’ was established. The model was prompted to extract core procedural information from UpToDate guidelines, generating a standardized nine-parameter objective checklist (identity, purpose, preparation, process, common risks, serious risks, post-procedural care, alarm symptoms, and alternatives). By comparing the actual consent forms against this AI-generated benchmark, the algorithm quantified information asymmetry and omission risks. A consensus text derived from three repeated sessions (a triple-check process via Gemini 3.1 Pro) ensured data consistency and minimized output variance. To evaluate these findings independently, the gap analysis outputs were subsequently cross-verified using the Anthropic Claude Sonnet 4.6 model.

### 2.4. Multidisciplinary Clinical Evaluation Cohort

A multidisciplinary cohort (*n* = 58) established the human baseline for Phase 1, collectively conducting 100 independent clinical evaluations across the five targeted procedures. An expert sub-cohort (*n* = 6; two public health specialists, three ethics committee members, and one hospital attorney) evaluated all five forms to assess institutional rhetoric and defensive medicine. A clinical sub-cohort of nineteen neurology residents assessed medical adequacy, readability, and time cost, generating 37 individual evaluations. A lay evaluation sub-cohort (*n* = 33 individuals without medical training) reported jargon-induced anxiety, cognitive retention, and subjective comprehension for a single randomly assigned form. All evaluations utilized standardized rubrics corresponding directly to the automated UHCI parameters, enabling a direct comparative analysis between human and AI performance (Figure 1B).

### 2.5. Architecture of the Universal Health Communication Index (UHCI) Algorithm

The UHCI algorithm comprises five submodules that analyze, in a multidimensional manner, the communicative quality and educational impact of medical consent forms on patients.

#### 2.5.1. Accessibility ($S_{\left\{ACC\right\}})$

This module is calculated out of 100 by weighting four different sub-parameters: Readability, Cognitive Load, Word Rarity, and Visual Design.

Syntactic Readability (*R*):

The structural readability of texts was measured using validated formulas and normalized between 0 and 100.

*For English Form (USA):* The normalized average of the Flesch–Kincaid Grade Level, Gunning Fog Index, and SMOG Index was used [12,18,19].

*For Turkish Form (TR):* The average of the Ateşman, and Çetinkaya–Uzun formulas, was used [20,21,22]. To confirm the automated readout of the Turkish cohort, the algorithm’s calculations were cross-verified using the independent web-based assessment tool ‘okunabilirlik.com’ [23].

Linguistic Complexity and Cognitive Load/Perplexity (*P*):

Beyond basic sentence structure, grammatical fluency and semantic unpredictability were measured using the Perplexity (PPL) metric derived from the autoregressive GPT-2 architecture. Within the computational pipeline, PPL calculations were executed via PyTorch (version 2.2, Linux Foundation, San Francisco, CA, USA) and Hugging Face Transformers (version 4.38, Hugging Face Inc., New York, NY, USA). While the generalized UHCI framework is designed around standard GPT-2 tokenization, the language-specific pre-trained checkpoint ‘ytu-ce-cosmos/turkish-gpt2’ was specifically loaded for the Phase 1 Turkish dataset to accurately process the assessment cohort (Figure 1B) [24].

Word Rarity/Zipf’s Law Analysis (*Z*):

Cognitive load was measured using Zipf’s Law-based word frequency analysis. According to the Dale–Chall concept, words outside the 3000 most frequently used were considered rare [25]. For English, the Brown Corpus [26] was used. For Turkish, Wordfreq database [27,28] was utilized. Because excessive medical jargon is a well-documented barrier to shared decision-making, if more than 15 percent of the words are rare, the accessibility score is reduced using a mathematical penalty. The rarity thresholds were verified through a custom NLP pipeline utilizing the ‘wordfreq’ library and ‘snowballstemmer’, which incorporated a typo-filtering mechanism to strictly exclude meaningless character strings [28,29].

Visual and Structural Design (*V*):

A five-point (0–4) structural Likert scale, adapted from PEMAT, was converted to a 100-point system by averaging scores from three independent AI Persona repetitions. To establish methodological reliability, these autonomous evaluations were directly correlated with human ground-truth scores provided by the clinical sub-cohort using the standard PEMAT rubric.

The accessibility score $S_{\left\{ACC\right\}}$ was obtained using the following formula:

$$S_{\left\{ACC\right\}}=\left(R\times 0.40\right)+\left(P\times 0.20\right)+\left(Z\times 0.20\right)+\left(V\times 0.20\right)\;$$

#### 2.5.2. Transparency ($S_{\left\{TRA\right\}})$

This module analyzes the capacity of consent forms to inform patients objectively while evaluating the unintended psychological stress this transparency might create. Balancing objective risk disclosure with emotional burden is a critical component of ethical, patient-centered communication. The module assesses this balance across four parameters: Medical Jargon, Risk Statement Density, Sentiment Analysis, and Textual Alarm Index.

Medical Jargon and Terminology Density (*J*):

Lists of “Unknown Words” flagged by the AI Persona were compiled, explicitly separating legal terminology (transferred to Section 2.5.4). English medical jargon was identified via an NER model pre-trained on SNOMED-CT and UMLS (Unified Medical Language System) [30,31]. Turkish texts underwent rule-based morphological screening. “Net Medical Jargon” combined AI Persona unknowns with system-identified terms [31]. Jargon ratios below 2 percent scored 100 points; those exceeding 15 percent scored 0. Outputs were cross-verified against a custom root-based NLP morphological screening tool.

Risk Statement Density (*D*):

Rule-based lexicon matching was used to quantify the explicit disclosure of clinical risks. While transparency is essential for patient autonomy, identifying whether documents genuinely inform patients—or rely on institutionally protective phrasing—is crucial for healthcare equity. Scoring penalizes forms omitting predefined risk words, thereby rewarding explicit and transparent risk disclosure (zero words: 20 points; one to two: 50 points; three to five: 80 points; six or more: 100 points). Computational accuracy was benchmarked using the GLiNER zero-shot NER model combined with Stanza-based chunking [32].

Sentiment Analysis (*E*):

To evaluate whether the document’s tone might induce disproportionate fear and deter patients from necessary clinical care, sentiment analysis was performed using BERT (Bidirectional Encoder Representations from Transformers) models with a language-specific trained bidirectional encoder architecture [33]. The ‘Probability of Negative Class’ (Softmax output) was directly converted to a score (Score = P(Negative) × 100). This neural output was cross-lingually benchmarked using a rule-based VADER (Valence Aware Dictionary and sEntiment Reasoner) analyzer on machine-translated sentences [34].

Textual Alarm Index (*X*):

The three-repeated average of the 1–10 Likert-type textual alarm scores generated by the “AI Persona” after reviewing the text was calculated and converted to a 100-point scale. This parameter evaluates the structural density of procedural risks, alarming terminology, and legal warnings contained in the consent text. To evaluate the communicative baseline of the documents, these automated textual alarm scores were descriptively compared against the subjective ratings reported by the lay evaluation sub-cohort using an identical 1–10 Likert scale.

The transparency score $S_{\left\{TRA\right\}}\;$ was obtained using the following formula:

$$S_{\left\{TRA\right\}}=\left(J\times 0.30\right)+\left(D\times 0.30\right)+\left(E\times 0.20\right)+\left(X\times 0.20\right)\;\;\;\;$$

#### 2.5.3. Autonomy ($S_{\left\{AUT\right\}})$

The Autonomy module evaluates the extent to which the consent form fosters communicative agency and supports Shared Decision-Making (SDM). By analyzing directive language, this module assesses whether the document reads as a collaborative medical choice or a compulsory institutional mandate. It is scored across three parameters: Semantic Pressure, Social Distance, and Syntactic Passivity.

Semantic Pressure and Option Analysis (*C*):

To quantify whether the document employs a directive, pressuring tone (which undermines patient autonomy) or an option-offering tone, a cross-lingual XLM-RoBERTa-based Zero-Shot Classification architecture was utilized [14]. XLM-RoBERTa has been previously validated for classifying pragmatic nuances in healthcare communication [10]. The model calculated “Option/Freedom” ($O_{\left\{score\right\}}$) and “Pressure/Obligation” ($P_{\left\{score\right\}}$) probability values for each sentence. If the pressure score $P_{\left\{score\right\}}\;$ outweighs the option score ($O_{\left\{score\right\}}$) the score approaches 0 logarithmically; if option-offering expressions are predominant, the score asymptotically approaches 100. To ensure methodological reliability, and avoid architectural bias, the core XLM-RoBERTa classification was cross-verified using an independent classifier (mDeBERTa-v3-base-mnli-xnli) based on a Linguistic Inquiry and Word Count (LIWC)-inspired benchmark; clinically, these scores were correlated with evaluations of institutional authority provided by the expert sub-cohort (*n* = 6) [35].

Social Distance/Pronoun Analysis (*I*):

The psychological distance and power dynamic between the institution and the patient were analyzed using “Pronoun Frequency Analysis.” In clinical communication, an overreliance on institutional pronouns can marginalize the patient’s active role. The total number of words rooted in first-person singular “I/Me” in the text is defined as $N_{\left\{Individual\right\}}$, while the total number of words rooted in plural or second-person (“We/You”) is defined as $N_{\left\{Authority\right\}}$. The Social Distance Ratio $R_{\left\{social\right\}}$, is calculated using the following equation:

$$R_{social}=\frac{N_{individual}}{N_{individual}+\;N_{authority}}$$

The obtained ratio has been linearly mapped to a 100-point scale. This metric was computationally verified using morphological parsers to track explicit pronouns and hidden subjects, and clinically corroborated by ground-truth evaluations from the expert sub-cohort (*n* = 6) [36].

Syntactic Passivity/Passive Voice Density (*U*):

Passive voice often obscures clinical responsibility and diminishes patient agency by removing the active ‘actor’ from the sentence. Target language-specific syntactical parsers were used to measure the density of these passive structures. Based on Shared Decision-Making communication models in the literature [37], a passive sentence ratio below 10% was rewarded with 100 points, while a ratio exceeding 50% resulted in 0 points. This metric was computationally verified using the Stanza NLP library to structurally isolate verbs and identify passive grammatical voices (‘Voice = Pass’) [36].

The autonomy score $S_{\left\{AUT\right\}}$ was obtained using the following formula:

$$S_{\left\{AUT\right\}}=\left(C\times 0.40\right)+\left(I\times 0.30\right)+\left(U\times 0.30\right)$$

#### 2.5.4. System Burden ($S_{\left\{BUR\right\}})$

The System Burden module evaluates how a consent form impacts operational efficiency and outpatient clinical workflows. Aligning with the need to assess AI tools based on their real-world applicability rather than mere textual accuracy [11], this module measures the systemic load caused by defensive medicine and excessive information volume. When a consent document prioritizes institutional liability protection (the “Legal Shield”) over patient comprehension, it generates structural inefficiencies and communication barriers. Therefore, the Legal Terminology Density is weighted at 40%. Practical barriers that directly obstruct the workflow, specifically Simulated Clinical Time Cost and Textual Volume, share the remaining 60% equally.

Legal Terminology Density and Institutional Discourse Index (*L*):

Forms are analyzed using legal lexicons from Module 2.1. A legal terminology ratio below 1.0 percent reflects patient-focused transparency (100 points), while a ratio above 5.0 percent indicates high structural information asymmetry and institutional dominance (0 points). To ensure reliability, the system’s outputs were computationally benchmarked using the ‘mDeBERTa-v3’ model to categorize sentence-level defensive medicine rhetoric [38].

Simulated Clinical Time Cost (*T*):

To reflect true cognitive recall limits rather than casual reading speeds, reading thresholds were set at 130 words per minute (WPM) for analytic languages (English) and adjusted to 100 WPM for agglutinative languages (Turkish) due to their morphological complexity and longer average word lengths. Meta-analytic data demonstrates that reading tasks requiring memory recall and comprehension drop reading speeds from a casual ~200 WPM down to 122–130 WPM [39,40]. The average duration of three AI persona repetitions determines “Additional Communication Time,” assuming 2.5 min per major question. If the total time is under 15 min, the form scores 100 points; over 30 min, it scores zero. To establish clinical alignment, the AI-simulated time cost was directly correlated with human ground-truth data, combining actual reading durations from the lay evaluation sub-cohort with estimated clinical explanation times from the clinical sub-cohort.

Textual Volume and Information Load (*W*):

Excessively long forms increase the risk that patients will sign without reading, leading to cognitive fatigue [41]. Forms below the ideal focus limit of 1000 words received full points; a geometric penalty system was applied, deducting one point (−1) for every 50-word block exceeding 1000 words. Textual volume and word count measurements were verified using the independent web-based assessment tool ‘okunabilirlik.com’ [23].

The system Burden score $S_{\left\{BUR\right\}}$ was obtained using the following formula:

$$S_{\left\{BUR\right\}}=\left(L\times 0.40\right)+\left(T\times 0.30\right)+\left(W\times 0.30\right)$$

#### 2.5.5. Medical Adequacy ($S_{\left\{MED\right\}})$

The Medical Adequacy module evaluates whether the consent form fulfills its primary clinical purpose: accurately conveying essential medical information to ensure patient safety and enable truly informed consent. It measures clinical accuracy and the resulting cognitive retention across three parameters.

Semantic Gold Standard Compliance (*G*):

Using the UpToDate reference database, ‘AI Gold Standard’ texts were classified into nine clinical categories. Semantic overlap was determined in vector space using cosine similarity [15] with a multilingual Transformer model (paraphrase-multilingual-MiniLM-L12-v2) [15]. To allow for natural linguistic variation while ensuring strong semantic equivalence, the Semantic Textual Similarity threshold was set at >0.60, a standard baseline in applied NLP for sentence-level paraphrase detection. If the match rate for the ‘Serious Risks’ category was below 50 percent, the system imposed an algorithmic cross-penalty on the Transparency score. To establish reliability, the algorithm’s semantic compliance was benchmarked using the Anthropic Claude Sonnet 4.6 LLM, and corroborated by ground-truth scores from the clinical sub-cohort.

Communication Gap Analysis (*Q*):

Identifying unaddressed patient concerns is vital for closing the communication gap prior to a procedure. The questions asked by the “AI Persona” simulation were matched to Gold Standard categories. If the question omitted by the form fell into a critical category, a heavy penalty (−20 points) was applied; if it fell into a standard category, a light penalty (−5 points) was applied. The identification of these omitted categories was verified using targeted evaluations via the Claude Sonnet 4.6 model.

Cognitive Retention (*K*):

True informed consent requires the patient to remember critical safety information after the consultation. Therefore, the retention output generated by the ‘AI Persona’ was compared in vector space with UpToDate’s ‘Serious Complications’ and ‘Alarm Symptoms’ pool. If the AI Persona correctly retained at least one critical risk (cosine similarity > 0.60), it received 100 points. To confirm cognitive alignment, these autonomous retention outputs were cross-referenced with actual information recall data reported by the lay evaluation sub-cohort.

The Medical Adequacy score $S_{\left\{MED\right\}}\;$ was obtained using the following formula:

$$S_{\left\{MED\right\}}=\left(G\times 0.40\right)+\left(Q\times 0.30\right)+\left(K\times 0.30\right)$$

### 2.6. Calculation of the UHCI Score

Raw scores from five independent modules were algorithmically integrated to generate the final UHCI score. A cross-penalty system was implemented to balance strict transparency rules with the pragmatic requirements of clinical and legal applicability (clinical realism). The contribution of each module to the final score was weighted according to established principles of patient autonomy [42] and disclosure of material risks [43]. Completeness of Medical Adequacy was assigned the highest weight (30%) to prioritize patient safety, followed by Autonomy (20%) and Transparency (20%). Finally, Accessibility (15%) and System Burden (15%) were included as operational coefficients, representing the practical workflow mechanisms of information delivery.

Furthermore, the structural penalties applied within these submodules (such as penalizing passive voice and medical/legal jargon) are grounded in established health literacy frameworks, including the Federal Plain Language Guidelines and the CDC Clear Communication Index [44,45]. To operationalize these qualitative guidelines into an automated quantitative tool, heuristic numeric cutoffs were established (e.g., linearly scaling the passive voice penalty from 10% to 50%).

The total score ${UHCI}_{\left\{Total\right\}}$ was calculated using the following formula:

$${UHCI}_{\left\{Total\right\}}=\left(S_{\left\{ACC\right\}}\times 0.15\right)+\left(S_{\left\{TRA\right\}}\times 0.20\right)+\left(S_{\left\{AUT\right\}}\times 0.20\right)+\left(S_{\left\{BUR\right\}}\times 0.15\right)+\left(S_{\left\{MED\right\}}\times 0.30\right)$$

### 2.7. Statistical Analysis

Statistical analyses for the clinical preliminary field assessment phase were performed using IBM SPSS Statistics (Version 28.0) and Python 3 (Pingouin statistical package). The normality of the data distribution was assessed using the Shapiro–Wilk test. To highlight the significant variance in human evaluations, continuous and scoring variables were summarized using means, standard deviations (SD), and minimum-maximum ranges. Furthermore, to mathematically quantify the inter-rater reliability and subjective variance among the human evaluators, the Intraclass Correlation Coefficient (ICC; two-way mixed, absolute agreement, average measures) was calculated.

Given the exploratory nature and the small document sample size (*N* = 5 consent forms), descriptive rank relationships were examined using Spearman’s rank correlation coefficient. These analyses are reported strictly as preliminary exploratory observations rather than formal inferential hypothesis testing. Mean differences and standard deviations were calculated to provide initial descriptive benchmarks. Finally, to evaluate the structural robustness of the final UHCI score against the designated heuristic weights (30/20/20/15/15), a sensitivity analysis was conducted. An alternative ‘Equal-Weight Scenario’ (assigning a flat 20% to all five sub-modules) was calculated to measure the percentage of score variance and confirm the stability of the index.

## 3. Results

### 3.1. Human Expert Evaluations and Feasibility Analysis

#### 3.1.1. Multidisciplinary Preliminary Feasibility (Phase 1)

Table 1 presents descriptive comparisons between the autonomous UHCI scores and human evaluations (*n* = 58). Spearman’s rank coefficients are provided to illustrate general alignment trends, such as the inverse rank relationship for System Burden (ρ = −0.975) and the positive alignment for Autonomy and Total Score (ρ = 0.600). Given the constrained document sample size (*N* = 5), these coefficients are presented as exploratory observations. They serve as preliminary indicators of the algorithm’s operational feasibility, without asserting formal inferential statistical significance. To address the difference between algorithmic outputs and human perception, a descriptive comparison of raw scores was conducted between the AI Textual Alarm Index (median of three iterations) and the subjective ratings of the lay cohort (*n* = 33). For high-risk, acute procedures, the AI index aligned closely with human perception (tPA: AI 9.0 vs. Lay 8.0; PE: AI 9.0 vs. Lay 7.0). However, a marked divergence was observed in lower-risk procedures. For the botulinum toxin form, lay evaluators reported a median score of 2.0, whereas the AI scored the structural risk density at 7.3. This gap demonstrates that the AI does not simulate subjective human emotion or contextual risk filtering; rather, it acts as a strict auditor of alarming medical and legal terminology.

Furthermore, significant divergences were observed in specific modules, particularly Transparency (ρ = −0.500) and Accessibility (ρ = 0.100). Notably, large numerical gaps emerged in individual assessments; for instance, the algorithm scored the Transparency of the Botulinum Toxin form at 51.34, whereas the human cohort scored it at 90.02. Similarly, the algorithm reached the maximum score ceiling (100.0) for System Burden in two forms, while human evaluations remained between 54.10 and 65.12. These disagreements highlight a structural divergence between human cognitive evaluation and algorithmic rule-based auditing, which warrants further mechanistic analysis. Therefore, the close alignment observed in the Total Score is a result of mathematical aggregation and does not reflect agreement at the individual module level.

#### 3.1.2. Descriptive Alignment and the Lack of a Human Gold Standard

A Bland–Altman descriptive analysis mapped the overall mean deviation (bias) between the autonomous UHCI outputs and the human baseline aggregates at +2.73 points. However, due to the limited procedural sample size (*N* = 5), formal limits of agreement cannot establish absolute statistical equivalence. Furthermore, as detailed in Table 2, the human assessment cohort exhibited high inter-rater variability and wide scoring dispersion across all modules (e.g., an SD of 31.21 in expert legal density evaluations). Inter-rater reliability was calculated where the dataset permitted (ICC, two-way mixed, absolute agreement, average measures). The Expert cohort demonstrated moderate agreement in evaluating legal and defensive tone (ICC = 0.60), reflecting observable variance even among specialists. For the Resident cohort, a traditional ICC could not be computed due to the sparse, unbalanced nature of the assessment design (where each resident evaluated 1–2 random forms to simulate real-world workflow). However, the extreme individual variance in subjective scores (ranging from 5 to 100) reflects demonstrably poor inter-rater agreement.

### 3.2. Cross-Lingual Proof of Concept and Case Study (Phase 2)

**Figure 2:**
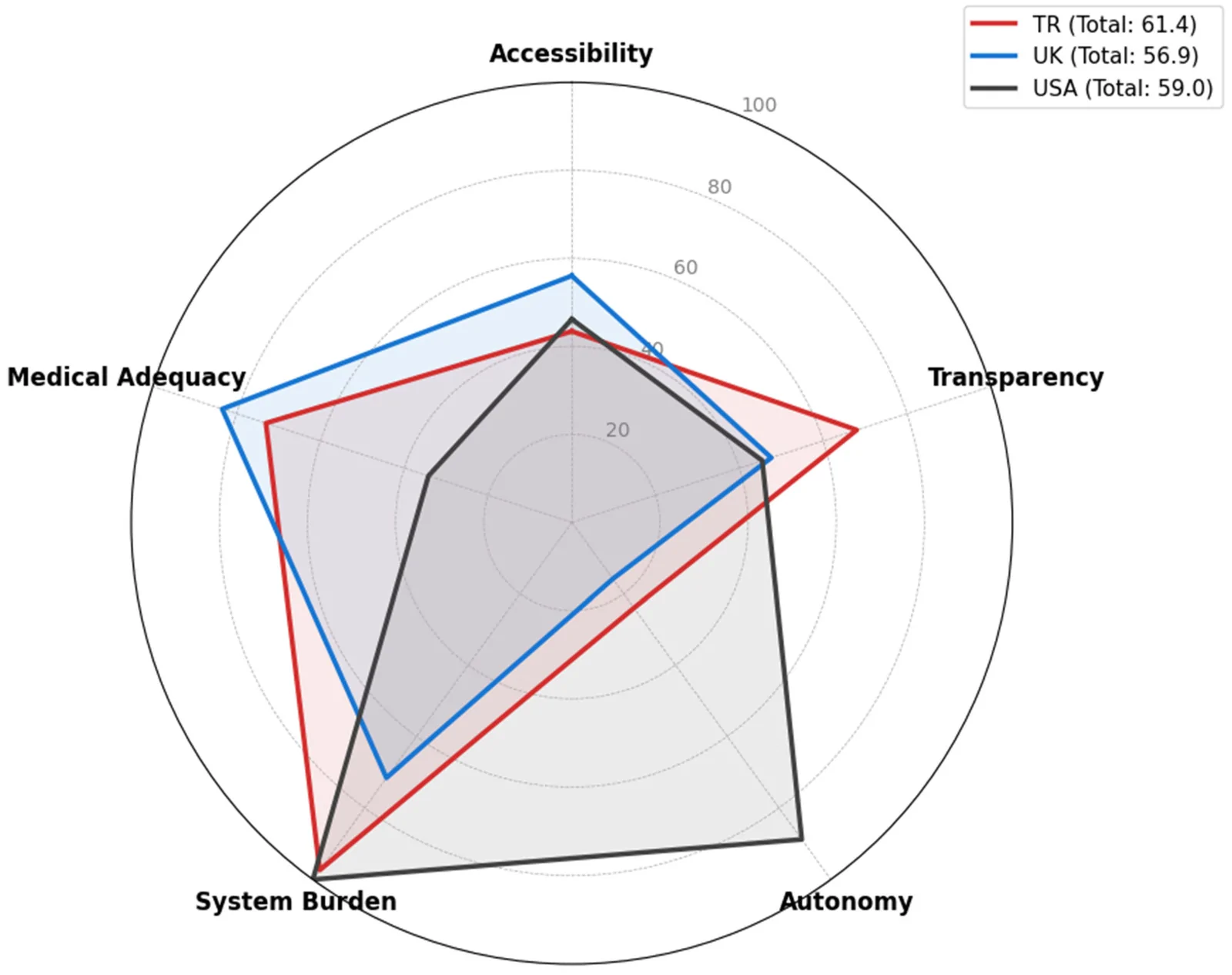
Multidimensional bioethical footprints and overlaid UHCI radar chart distributions of Lumbar Puncture (LP) consent forms across three distinct healthcare systems. The radar chart illustrates how comparable global UHCI scores can mask deep sub-modular variations in institutional communication strategies for the exact same clinical procedure. The USA form displays a structural emphasis on patient Autonomy but demonstrates a severe deficit in Medical Adequacy. The UK form exhibits strong Medical Adequacy but shows significant deficits in Autonomy due to directive phrasing. The Turkish (TR) form presents a pronounced peak in the System Burden module, reflecting higher systemic complexity. This controlled cross-lingual comparison underscores the index’s ability to objectively distinguish between competing institutional health policies—specifically whether a system prioritizes patient-centered autonomy, comprehensive medical disclosure, or institutional risk aversion.

To demonstrate cross-lingual applicability, under strict methodological control, three lumbar puncture (LP) consent forms from distinct healthcare systems (Turkey, UK, USA) were analyzed. The global UHCI scores were remarkably comparable: 61.41 for TR, 56.86 for UK, and 59.03 for USA. However, their sub-module distributions differed drastically, highlighting contrasting institutional policies (Figure 2). The USA form demonstrated exceptionally high Autonomy (88.78) but failed in Medical Adequacy (34.21) due to the omission of critical procedural risks. Conversely, the UK NHS form exhibited strong Medical Adequacy (83.43) but severe deficits in Autonomy (15.84) due to high passive voice density (41.3%) and directive phrasing. The Turkish form presented the highest System Burden (97.35). This controlled comparison proves the algorithm’s capacity to detect contrasting communication strategies across global health contexts. Granular parameters are detailed in Table 3.

### 3.3. Robustness and Sensitivity Analysis

To empirically evaluate the impact of the heuristic weights on the final UHCI score, a sensitivity analysis was conducted on the cross-lingual dataset using an ‘Equal-Weight Scenario’ (20% per module).

The analysis demonstrated that absolute total scores shifted within a narrow margin under equal weighting. The Turkish (TR) form showed negligible variance (−0.43%, shifting from 61.41 to 61.15), while the UK form decreased slightly (−3.45%, from 56.86 to 54.90). The highest variance was observed in the USA form, which increased by 6.59% (from 59.03 to 62.92) when the heavier 30% weight on Medical Adequacy was neutralized.

## 4. Discussion

### 4.1. General Summary and Key Findings

This study details the development and preliminary feasibility demonstration of the Universal Health Communication Index (UHCI), an AI-driven Health Technology Assessment (HTA) framework evaluating the ethical, linguistic, and clinical quality of medical consent forms. Evaluated against a multidisciplinary human baseline (*n* = 58) and tested in a cross-lingual proof-of-concept, the system provided a reproducible evaluation baseline. The primary outcome is that bioethical and legal standards can be operationalized into a reproducible HTA tool. A critical observation from the human assessment phase was the stark inter-rater variance. While experts showed moderate consistency (ICC: 0.60), medical residents exhibited extreme individual disagreement. This divergence accurately reflects the heterogeneity of a standard clinical workforce, where detecting critical omissions (e.g., missing alternatives) relies heavily on a physician’s seniority. The extreme variability demonstrates that human evaluation of consent forms is inherently subjective and unreliable. Consequently, the UHCI is not designed to mimic this fluctuating human baseline. Instead, it anchors its assessments to objective clinical guidelines (e.g., UpToDate), providing a standardized, deterministic safety net that performs consistently regardless of human subjectivity

Crucially, the UHCI evaluates strictly the documents offered to patients, not the broader Shared Decision-Making (SDM) process itself. This AI-assisted screening identifies communicational and systemic barriers that obstruct SDM. By doing so, it provides health leaders and policymakers with actionable insights to optimize clinical workflows, support equitable patient engagement, and facilitate the responsible integration of AI into healthcare systems.

### 4.2. Implications for Theory: Transition from Structural Analysis to Bioethical Transparency

Most existing research evaluating the quality of consent forms has focused primarily on syntactic readability indices, such as Flesch–Kincaid and SMOG [12,19,46]. While recent NLP studies improve text comprehensibility, they fail to quantitatively measure systemic issues like “directive institutional discourse” and “information asymmetry [47]. Furthermore, a significant gap persists between qualitative LLM guidelines [48]. and quantitative HTA models. The UHCI addresses this gap by operationalizing these ethical dimensions to support evidence-based health policy.

In Sentiment and Autonomy analyses (Modules 2.5.2 and 2.5.3), standard NLP assumptions were deliberately reversed. Unlike typical sentiment analysis that generally rewards “positive” language, the UHCI penalizes overly positive texts if they mask directive, institution-centric biases that undermine patient autonomy and authentic shared decision-making. Additionally, passive voice usage—which frames the patient as an “object” rather than an active participant—has been mathematically quantified as a syntactic autonomy violation [49].

A key theoretical contribution of this study is advancing document assessment into a practical HTA framework by synthesizing LLM comprehension with established medical law—specifically the Montgomery v Lanarkshire ruling on the “disclosure of material risks” [50,51]. The UHCI addresses the “illusion of transparency” through a cross-security barrier between Medical Adequacy (Module 2.5.5) and Transparency (Module 2.5.2). If critical risks (e.g., death, paralysis) are structurally legible but semantically omitted compared to reference texts (UpToDate), the system algorithmically penalizes the form. By proactively flagging these informational omissions, the UHCI translates bioethical principles into a scalable screening mechanism. This approach demonstrates how AI can be practically integrated into Electronic Health Records (EHRs) and hospital workflows to support clinical governance, risk management, and health policy implementation.

The observed scoring disagreements between the algorithm and the human baseline reveal critical insights into the mechanics of clinical text evaluation. The large numerical gaps in Transparency (e.g., AI: 51.34 vs. Human: 90.02 for the Botulinum Toxin form) occur because human evaluators often conflate syntactic simplicity with bioethical transparency. A short, readable form feels ‘transparent’ to a human, yet the algorithm’s Risk Statement Density parameter strictly penalizes the form if explicit medical risks are omitted, thereby exposing the ‘illusion of transparency.’

Similarly, the maximum ceiling (100.0) reached by the algorithm for System Burden—while human evaluations remained significantly lower (54.10–65.12)—is driven by methodological divergences in measuring cognitive and legal friction. During the human baseline evaluation, patients reported very few questions (averaging 1–2 per form). In the context of complex medical texts, such low query rates often reflect ‘survey fatigue’ or the ‘illusion of comprehension’—a phenomenon extensively documented in the health literacy literature, where patients frequently underreport confusion due to social desirability or cognitive overload [52,53]. This clinical dynamic artificially deflates the human time-cost baseline. Uninfluenced by social desirability, the AI consistently generated multiple relevant questions (averaging 4–5) based on strict semantic complexity. Furthermore, while human experts evaluated legal terminology holistically via a 0–100 Visual Analog Scale (VAS)—cognitively normalizing daily institutional jargon—the algorithm relied on rigid lexical counting of defensive terms. Thus, the algorithm serves as an absolute, objective quantifier of systemic friction, whereas the human baseline reflects a normalized and subjective clinical tolerance.

### 4.3. Implications for Health Policy, Practice, and Clinical Governance

By quantifying the operational burden of defensive medicine, this study provides actionable insights for healthcare administration. The System Burden (Module 2.5.4) parameter measures this inefficiency through ‘Simulated Clinical Time Cost,’ assessing a form’s suitability for time-constrained outpatient settings. By calculating reading and interaction times, the UHCI provides ethics committees with an analytical feedback mechanism to optimize forms. This ensures they present medical risks comprehensively without imposing unnecessary health economic burdens, directly aligning with HTA priorities to support evidence-based health policies and health systems optimization [11].

To address the translational gap in health informatics, the UHCI is designed for seamless integration with Electronic Health Records (EHR) as a scalable Human-in-the-Loop (HITL) decision-support tool. Acting as a supportive HTA screening mechanism, it reflects successful multi-agent LLM orchestrations in health policy modeling [16]. While recent studies warn against AI hallucinations in clinical simulations [17], the UHCI mitigates this risk through UpToDate reference cross-verifications. Automating comprehensive textual reviews reduces cognitive and operational demands on administrators, facilitating the translation of computational advances into practical clinical governance [54,55].

Ultimately, the UHCI transitions the labor-intensive and highly subjective manual review of consent documents into a scalable, standardized process. By utilizing LLMs to rapidly screen documents across hospital networks or public health institutions, it can identify systemic communicational deficiencies. This scalability establishes a practical foundation for AI-assisted HTA modules to be integrated into national health policies and Hospital Information Management Systems, ultimately supporting digital health transformation and equitable patient care.

### 4.4. Study Limitations and Directions for Future Research

While this study introduces a practical HTA proof-of-concept, several limitations exist. First, the AI Persona operates strictly as a lexical auditor of textual risk density rather than a simulation of authentic human emotion; framing automated text analysis as true psychological anxiety introduces an anthropomorphic bias.

Second, the three-iteration protocol used for the AI Persona and AI Gold Standard served solely as a baseline filter for severe hallucinations. Three iterations are statistically insufficient to capture the full ideational variance and stochastic behavior of large language models, and future studies must employ larger sampling protocols to ensure prompt stability.

Third, the document corpus analyzed in this preliminary feasibility study was limited to five representative neurological informed consent forms (*N* = 5). Although evaluated across a 58-member human cohort, this constrained document sample limits the statistical power of formal inferential testing and restricts the generalizability of the correlation analyses. The reported correlation coefficients and mean differences must therefore be interpreted strictly as exploratory proof-of-concept metrics. Future multi-center studies with substantially larger document corpora across diverse clinical specialties (such as oncology or cardiovascular surgery) are required to establish normative statistical psychometrics. Additionally, while currently optimized for analytic (English) and agglutinative (Turkish) languages, adapting the algorithm for character-based linguistic structures and low-resource languages remains a critical future milestone.

Fourth, the reliance on a composite Total Score introduces a mathematical ‘cancellation effect’. Because the algorithm and human evaluators score specific dimensions differently, large differences in individual modules (such as System Burden and Transparency) can mathematically cancel each other out in the final sum. Therefore, a closely aligned Total Score is only a general summary and does not imply that the algorithm and human evaluators agree at the individual module level.

Furthermore, the weighting coefficients for the UHCI modules (e.g., 30 percent for Medical Adequacy) were derived from heuristic consensus and the bioethical literature. While the sensitivity analysis (Section 3.3) confirms the clinical necessity of this heuristic structure, these weights require future empirical optimization. For instance, under equal weighting, the score of the USA form is artificially inflated by over 6%, effectively masking its critical omission of procedural risks. By assigning a higher weight (30%) to Medical Adequacy, the original UHCI appropriately acts as a clinical safety net, preventing forms from achieving high overall scores through aggressive Autonomy metrics alone while compromising basic medical disclosure. Ultimately, deploying the UHCI across hospital networks to correlate AI-simulated cognitive load with real-world time-cost data will be critical in establishing its definitive operational utility as a routine clinical governance tool. Finally, while the algorithm’s penalties are fundamentally rooted in authoritative guidelines (e.g., CDC and Plain Language), the exact numeric thresholds used (such as the 10% limit for passive voice or 0.60 for cosine similarity) function as clinically-derived heuristic baselines. They are not absolute statistical cut-offs derived from ROC curve analyses of the patient data. For instance, there is no empirical data in this current study proving why the passive voice limit should be exactly 10% instead of 13%. Future studies using large-scale machine learning datasets are required to statistically optimize these specific numeric boundaries.

## 5. Conclusions

The Universal Health Communication Index (UHCI) translates the abstract bioethical and legal dimensions of informed consent into a quantifiable and scalable Health Technology Assessment (HTA) framework. By systematically evaluating clinical adequacy, information asymmetry, and the operational burden of defensive medicine, this preliminary proof-of-concept provides healthcare administrators and policymakers with a practical tool to optimize clinical documentation. Ultimately, the UHCI establishes a reproducible computational baseline for AI-assisted textual screening, demonstrating how AI can be responsibly integrated into health systems. It supports evidence-based health policies, fosters equitable shared decision-making, and enhances clinical workflows—strictly as a supportive mechanism, without replacing human clinical judgment.

## Figures and Tables

**Table 1:** Comparative Multi-Dimensional Performance and Statistical Alignment of the UHCI Algorithm vs. Human Baseline.

Dimension	tPA (U/H)	PE (U/H)	BTA (U/H)	EMG (U/H)	LP (U/H)	Spearman (ρ)	Academic/Clinical Interpretation
Accessibility	44.36/57.63	43.73/51.74	44.20/50.14	43.68/51.14	43.81/50.45	0.100	Weak Rank Alignment
Transparency	63.54/77.30	68.79/81.62	51.34/90.02	61.00/84.65	68.00/85.14	−0.500	Negative Trend
Autonomy	44.23/61.73	78.22/59.58	38.95/54.79	40.40/39.47	23.97/50.15	0.600	Positive Rank Alignment (ρ = 0.60)
System Burden	100.0/65.12	100.0/54.10	90.52/82.15	95.86/80.30	97.36/77.32	−0.975	Negative Rank Alignment
Medical Adequacy	70.58/47.39	74.00/56.98	77.88/53.82	57.32/51.37	73.00/42.13	0.500	Moderate-Strong Positive Trend
TOTAL SCORE	64.38/60.44	73.16/61.21	61.63/64.95	58.41/59.95	61.47/58.86	0.600 †	Clinical Agreement & Narrow Bias Range

Note: Scores are presented as UHCI (U)/Human Baseline (H) format. All dimensions and the TOTAL SCORE are evaluated on a standardized continuous scale ranging from 0 to 100, where higher scores reflect superior communicative quality. The exception is System Burden, where higher scores indicate a greater density of institutional defensive medicine elements. The Human Baseline represents the mean aggregate scores from the 58-member multidisciplinary assessment cohort. Spearman’s rank correlation (ρ) evaluates the monotonic trend and dimensional alignment across procedures. Due to the sample size constraint inherent in this proof-of-concept phase (N = 5 procedures), non-parametric methods were utilized; therefore, robust correlational trends may not reach the standard threshold for statistical significance. (†) Due to the N = 5 sample size limitation, formal limits of agreement cannot be statistically powered. The Bland–Altman metrics are presented strictly as a descriptive measure of the overall mean aggregate deviation (+2.73 bias), rather than absolute clinical equivalence. Abbreviations: BTA: Botulinum Toxin Application, EMG: Electromyography, LP: Lumbar Puncture, PE: Plasma Exchange, tPA: Tissue Plasminogen Activator, UHCI: Universal Health Communication Index.

**Table 2:** Descriptive Analysis of Human Evaluation Sub-Cohorts Across Standardized Consent Forms.

	Consent Forms	Median	Standard Deviation	Min–Max
experts (*N* = 6) Legal Terminology Density (0–100)	tPA (*n* = 6)	73.83	31.21	15–100
	EMG (*n* = 6)	33	27.96	0–80
	BTA (*n* = 6)	43.17	18.33	10–65
	PE (*n* = 6)	55.17	29.51	10–85
	LP (*n* = 6)	49.83	23.73	10–80
residents (*N* = 19) Medical Adequacy (0–100)	tPA (*n* = 7)	90	21.2	40–100
	EMG (*n* = 7)	75	24.8	25–100
	BTA (*n* = 8)	80	30.7	5–100
	PE (*n* = 7)	70	26.4	12–90
	LP (*n* = 8)	85	21.9	24–90
lay evaluators (*N* = 33) Anxiety (1–10)	tPA (*n* = 7)	8	2.1	5–10
	EMG (*n* = 5)	4	2.6	3–8
	BTA (*n* = 7)	2	2,6	1–7
	PE (*n* = 7)	6,5	3.6	1–10
	LP (*n* = 7)	5	2.6	3–10

Note: *N* represents the number of human evaluators within each designated sub-cohort. *n* represents the number of consent forms. The Expert cohort (consisting of public health specialists, an ethics committee member, and a hospital attorney) evaluated institutional risk aversion and defensive medicine metrics. The Resident cohort evaluated clinical accuracy, reading workload, and comprehensiveness. The Lay Evaluator cohort (non-medical participants) reported subjective cognitive retention and psychometric anxiety utilizing a standardized 1–10 Likert scale. Continuous variables are expressed as medians and ranges to account for non-parametric score distributions. Abbreviations: tPA: Tissue Plasminogen Activator, EMG: Electromyography, BTA: Botulinum Toxin Application, PE: Plasma Exchange, LP: Lumbar Puncture.

**Table 3:** Controlled Cross-Lingual Comparison: Granular Output and Sub-Parameter Breakdown of UHCI Modules for Lumbar Puncture (TR, UK, USA).

Module and Sub-Parameters	USA (Lumbar Puncture)	TR (Lumbar Puncture)	UK (Lumbar Puncture)
1. ACCESSIBILITY (ACC) OVERALL SCORE—Weight: 15%	46.18	43.43	56.04
Syntactic Readability Formulas (Weight: 40%)			
Flesch–Kincaid Grade (USA)/Ateşman (TR)	Raw: 15.2	Raw: 42.1	Raw: 10.5
Gunning Fog Index (USA)	Raw: 18.8	-	Raw: 13.0
SMOG Index (USA)/Çetinkaya–Uzun (TR)	Raw: 17.2	Raw: 28.4	Raw: 12.8
Normalized Readability Base Score (Mean)	→ Score: 2.9/100	→ Score: 35.2/100	→ Score: 27.1/100
Linguistic Complexity/GPT-2 Perplexity (Weight: 20%)	Raw PPL: 16.87 → Score: 51.51	Raw PPL: 63.97 → Score: 36.66	Raw PPL: 16.24 → Score: 51.98
Word Rarity/Zipf’s Law Ratio (Weight: 20%)	Ratio: 19.4% → Score: 100	Ratio: 49.7% → Score: 50.7	Ratio: 18.6% → Score: 100
Visual & Structural Design/Adapted PEMAT (Weight: 20%)	→ Score: 50.0/100	→ Score: 50.0/100	→ Score: 58.3/100
2. TRANSPARENCY (TRA) OVERALL SCORE—Weight: 20%	45.44 *	68.00 *	47.60 *
Medical Jargon Density (Weight: 30%)	Ratio: 2.2% (11 terms) → Score: 98.6	Ratio: 1.3% (9 terms) → Score: 100.0	Ratio: 1.2% (24 terms) → Score: 100.0
Risk Statement Volume (Weight: 30%)	4 terms detected → Score: 50.0	5 terms detected → Score: 80.0	3 terms detected → Score: 80.0
NLP Sentiment Score (Weight: 20%)	→ Score: 40.0/100	→ Score: 50.0/100	→ Score: 40.0/100
Textual Alarm Index (Weight: 20%)	→ Score: 16.7/100	→ Score: 20.0/100	→ Score: 30.0/100
Autonomous Cross-Penalty (Critical Risk)	Triggered (-%30)	Not Triggered (0%)	Triggered (-%30)
3. AUTONOMY(AUT) OVERALL SCORE—Weight: 20%	88.78	23.97	15.84
Semantic Pressure/Choice Score (Weight: 40%)	→ Score: 99.5/100	→ Score: 9.4/100	→ Score: 4.1/100
Social Distance/Pronoun Ratio (Weight: 30%)	Ratio: 0.74 (I: 32, We/You: 10) → Score: 100.0	Ratio: 0.00 (I: 0, We/You: 3) → Score: 0.0	Ratio: 0.17 (I: 22, We/You: 108) → Score: 33.6
Syntactic Passivity/Passive Voice Ratio (Weight: 30%)	Ratio: 27.6% → Score: 56.0	Ratio: 19.1% → Score: 77.1	Ratio: 41.3% → Score: 21.6
Autonomous Cross-Penalty Applied	None (Alternatives Presented)	None (Alternatives Presented)	None (Alternatives Presented)
4. SYSTEM BURDEN (BUR) OVERALL SCORE—Weight: 15%	100	97.38	71.59
Legal/Defensive Shield (Weight: 40%)	1 term (0.2%) → Score: 100.0	0 terms (0.0%) → Score: 100.0	0 terms (0.0%) → Score: 100.0
Simulated Clinical Time Cost (Weight: 30%)	Time: 13.1 min → Score: 100.0	Time: 16.3 min → Score: 91.2	Time: 26.4 min → Score: 23.7
Textual Volume/Information Load (Weight: 30%)	Vol: 505 words → Score: 100.0	Vol: 716 words → Score: 100.0	Vol: 1921 words → Score: 81.6
5. MEDICAL ADEQUACY (MED) OVERALL SCORE —Weight: 30%	34.21	73.00	83.43
Gold Standard Match/UpToDate (Weight: 40%)	→ Score: 14.0/100	→ Score: 62.5/100	→ Score: 66.1/100
AI Communication Gap (Weight: 30%)	→ Score: 75.0 (Penalty: −5)	→ Score: 60.0 (Penalty: −40)	→ Score: 90.0 (Penalty: −10)
Cognitive Retention (Weight: 30%)	→ Score: 0.0 (Failed)	→ Score: 100.0 (Successful)	→ Score: 100.0 (Successful)
TOTAL UHCI SCORE	59.03	61.41	56.86

Note: The table demonstrates the algorithm’s high-resolution diagnostic capacity across different legal and linguistic frameworks under a controlled clinical scenario. Final sub-parameter scores are scaled from 0 to 100. Parentheses indicate the raw data or metrics parsed by the LLM core before normalization. (*) The autonomous cross-penalty for ‘Critical Risk Omitted’ was triggered for the UK and USA forms, reducing their Transparency scores due to their failure to meet the semantic similarity thresholds for adequate serious risk coverage in the Medical Adequacy module. (→) The arrow symbol denotes the computational transition from raw linguistic/statistical metrics to normalized scores (0–100 scale). Abbreviations: AI: Artificial Intelligence, LLM: Large Language Model, LP: Lumbar Puncture, NLP: Natural Language Processing, PPL: Perplexity, TR: Türkiye, UHCI: Universal Health Communication Index, USA: United States of America, UK: United Kingdom.

## Data Availability

The minimal dataset, including the anonymized human assessment cohort scores and the analyzed medical consent form texts supporting the findings of this study, is available as Supplementary Materials. To ensure methodological transparency and permit independent auditing, the isolated Python functions executing the core scoring logic and heuristic thresholds have also been provided as a Supplementary File (UHCI Core Scoring Logic). The complete web architecture and underlying proprietary LLM orchestration pipelines are omitted from the public repository to protect pending intellectual property and commercial rights. Further inquiries can be directed to the corresponding author.

## Supplementary Materials

The following supporting information can be downloaded at: https://www.mdpi.com/article/10.3390/healthcare14182956/s1, Document S1: Original text versions of the evaluated informed consent forms; Table S1: Survey evaluation dataset of residents, medical specialists, and lay participants (SPSS data); Dataset S1: Raw UHCI evaluation metrics and baseline scoring outputs; File S1: Algorithmic computation workflows and mathematical formulas of the UHCI framework.

## References

[1] Grady C. (2015). Enduring and Emerging Challenges of Informed Consent. N. Engl. J. Med..

[2] World Medical Association (2013). World Medical Association Declaration of Helsinki: Ethical Principles for Medical Research Involving Human Subjects. JAMA.

[3] U.S. Department of Health and Human Services (HHS) (2018). Title 45 Public Welfare, Part 46 Protection of Human Subjects (The Common Rule).

[4] van Delden J.J.M., van der Graaf R. (2017). Revised CIOMS International Ethical Guidelines for Health-Related Research Involving Humans. JAMA.

[5] Paasche-Orlow M.K., Taylor H.A., Brancati F.L. (2003). Readability Standards for Informed-Consent Forms as Compared with Actual Readability. N. Engl. J. Med..

[6] Krumholz H.M. (2010). Informed Consent to Promote Patient-Centered Care. JAMA.

[7] Wang C., Li M., He J., Wang Z., Darzi E., Chen Z., Ye J., Li T., Su Y., Ke J. (2025). A survey for large language models in biomedicine. Artif. Intell. Med..

[8] Wang H., Zhang J., Wu S., Wei H., Chen X., Ou Y., Sun X. (2025). MMSupcon: An image fusion-based multi-modal supervised contrastive method for brain tumor diagnosis. Artif. Intell. Med..

[9] Wang W., Ma L., Cai W., Zhao H., Zhang X. (2025). HMEA: A hierarchical medical knowledge graph entity alignment model fusing multi-aspect information. Artif. Intell. Med..

[10] Consolandi M., Magnolini S., Dragoni M. (2026). Risk Communication in Healthcare: The Management of Misunderstandings. J. Med. Syst..

[11] Simpao A.F. (2026). From Prototype to Production: Three Priorities for Journal of Medical Systems in 2026. J. Med. Syst..

[12] Flesch R. (1948). A new readability yardstick. J. Appl. Psychol..

[13] Shoemaker S.J., Wolf M.S., Brach C. (2014). Development of the Patient Education Materials Assessment Tool (PEMAT): A new measure of understandability and actionability for print and audiovisual patient information. Patient Educ. Couns..

[14] Conneau A., Khandelwal K., Goyal N., Chaudhary V., Wenzek G., Guzmán F., Grave E., Ott M., Zettlemoyer L., Stoyanov V., Jurafsky D., Chai J., Schluter N., Tetreault J. (2020). Unsupervised Cross-lingual Representation Learning at Scale. Proceedings of the 58th Annual Meeting of the Association for Computational Linguistics.

[15] Reimers N., Gurevych I., Inui K., Jiang J., Ng V., Wan X. (2019). Sentence-BERT: Sentence Embeddings using Siamese BERT-Networks. Proceedings of the 2019 Conference on Empirical Methods in Natural Language Processing and the 9th International Joint Conference on Natural Language Processing (EMNLP-IJCNLP).

[16] Deng Y., Dennstädt F., Filchenko I., van der Meer J., Yang X., Schmidt M.H., Bassetti C.L.A., Tzovara A., Denecke K. (2026). Comorbidity Classification from Clinical Free-Text using Large Language Models: Application to Sleep Disorder Patients. J. Med. Syst..

[17] Rios-Garcia W., Silva-Jiménez S., Gálvez-Rodríguez E., Alberca-Naira Y., Via-y-Rada-Torres A.D., Rios-Garcia A.A. (2026). Assessment of ChatGPT-5 as an Artificial Intelligence Tool for Exploring Emerging Dimensions of Clinical Simulation: A Proof-of-concept Study. J. Med. Syst..

[18] Gunning R. (1952). The Technique of Clear Writing.

[19] Mc Laughlin G.H. (1969). SMOG grading-a new readability formula. J. Read..

[20] Ateşman E. (1997). Türkçede okunabilirliğin ölçülmesi. Ank. Üniversitesi Tömer Dil. Derg..

[21] Bezirci B., Yılmaz A.E. (2010). Proposal of a New Readability Metric for Turkish. 2010 IEEE 18th Signal Processing and Communications Applications Conference.

[22] Çetinkaya G., Uzun G.L. (2012). Identifying and classifying the readability levels of Turkish texts. ATINER’S Conference Paper Series, No: LIT2012-0281.

[23] Türkçe Metinler İçin Okunabilirlik Analizi Aracı. https://okunabilirlik.com.

[24] YTU-CE-COSMOS (2023). Turkish-GPT2: A Generative Pre-Trained Transformer Model for Turkish. https://Huggingface.Co/Ytu-Ce-Cosmos/Turkish-Gpt2.

[25] Dale E., Chall J.S. (1948). A formula for predicting readability: Instructions. Educ. Res. Bull..

[26] Francis W.N. (1965). A standard corpus of edited present-day American English. Coll. Engl..

[27] Ortiz Suárez P.J., Romary L., Sagot B., Jurafsky D., Chai J., Schluter N., Tetreault J. (2020). A Monolingual Approach to Contextualized Word Embeddings for Mid-Resource Languages. Proceedings of the 58th Annual Meeting of the Association for Computational Linguistics.

[28] Speer R. (2022). Rspeer/Wordfreq: v3.0.

[29] Porter M.F. (2001). Snowball: A Language for Stemming Algorithms. http://snowball.tartarus.org/.

[30] Bodenreider O. (2004). The Unified Medical Language System (UMLS): Integrating biomedical terminology. Nucleic Acids Res..

[31] Lee J., Yoon W., Kim S., Kim D., Kim S., So C.H., Kang J. (2020). BioBERT: A pre-trained biomedical language representation model for biomedical text mining. Bioinformatics.

[32] Zaratiana U., Tomeh N., Holat P., Charnois T. (2023). GLiNER: Generalist Model for Named Entity Recognition Using Bidirectional Transformer.

[33] Devlin J., Chang M.-W., Lee K., Toutanova K. (2019). BERT: Pre-training of Deep Bidirectional Transformers for Language Understanding. Proceedings of the 2019 Conference of the North.

[34] Hutto C., Gilbert E. (2014). VADER: A Parsimonious Rule-based Model for Sentiment Analysis of Social Media Text. Proceedings of the Eighth International Conference on Weblogs and Social Media (ICWSM-14).

[35] Pennebaker J.W., Boyd R.L., Jordan K., Blackburn K. (2015). The Development and Psychometric Properties of LIWC2015.

[36] Qi P., Zhang Y., Zhang Y., Bolton J., Manning C.D. (2020). Stanza: A Python Natural Language Processing Toolkit for Many Human Languages.

[37] Elwyn G., Frosch D., Thomson R., Joseph-Williams N., Lloyd A., Kinnersley P., Cording E., Tomson D., Dodd C., Rollnick S. (2012). Shared Decision Making: A Model for Clinical Practice. J. Gen. Intern. Med..

[38] He P., Gao J., Chen W. (2023). DeBERTaV3: Improving DeBERTa using ELECTRA-Style Pre-Training with Gradient-Disentangled Embedding Sharing. arXiv.

[39] Brysbaert M. (2019). How many words do we read per minute? A review and meta-analysis of reading rate. J. Mem. Lang..

[40] Carver R.P. (1992). Reading Rate: Theory, Research, and Practical Implications. J. Read..

[41] Hochhauser M. (2004). Informed consent: Reading and understanding are not the same: Subjects may read consent forms, but they don’t always understand them. Appl. Clin. Trials.

[42] Beauchamp T., Childress J. (2019). Principles of Biomedical Ethics: Marking Its Fortieth Anniversary. Am. J. Bioeth..

[43] Appelbaum P.S. (2007). Clinical practice. Assessment of patients’ competence to consent to treatment. N. Engl. J. Med..

[44] Centers for Disease Control and Prevention (2014). The CDC Clear Communication Index.

[45] Plain Language Action and Information Network (2011). Federal Plain Language Guidelines.

[46] Sönmez L.Ö., Sönmez M.G., Ayrancı M.K., Gül M. (2018). Evaluation of the readability of informed consent forms used for emergency procedures. Disaster Emerg. Med. J..

[47] Koç U., Güneş Y.C., Çolakoğlu M.N., Coşkun N., Dere V., Özdemir M., Özdemir E. (2026). ChatGPT-generated informed consent forms in interventional radiology: A randomized controlled evaluation of comprehension, attitudinal responses, and readability. Eur. J. Radiol..

[48] Georgiou G.P. (2026). An AI-assisted framework for the ethical use of machine learning in healthcare. Int. J. Med. Inform..

[49] Rundblad G. (2007). Impersonal, General, and Social: The Use of Metonymy Versus Passive Voice in Medical Discourse. Writ. Commun..

[50] (2015). Montgomery v Lanarkshire Health Board [2015] UKSC 11.

[51] Spatz E.S., Krumholz H.M., Moulton B.W. (2016). The New Era of Informed Consent. JAMA.

[52] Street R.L., Gordon H.S., Ward M.M., Krupat E., Kravitz R.L. (2005). Patient Participation in Medical Consultations. Med. Care.

[53] Parikh N.S., Parker R.M., Nurss J.R., Baker D.W., Williams M.V. (1996). Shame and health literacy: The unspoken connection. Patient Educ. Couns..

[54] Olawade D.B., Plabon S.B., Ojo A., Ogunbona M.A., Makanjuola B.D., Olasilola O.R. (2026). Human in the loop artificial intelligence in healthcare: Applications, outcomes, and implementation challenges. Int. J. Med. Inform..

[55] Vijayasarathy S. (2026). From algorithmic innovation to clinical deployment: A systematic review of methodological gaps limiting federated learning in healthcare. Int. J. Med. Inform..

