# Supplementary material for: Autonomous Assessment of Medical Consent Forms: Development and Preliminary Feasibility Demonstration of the Universal Health Communication Index (UHCI)
Source: Healthcare (Basel). 2026 Sep 10;14(18):2956. doi: 10.3390/healthcare14182956 (PMC13606284; doi:10.3390/healthcare14182956)
Supplement: Supplementary file 1 [file healthcare-14-02956-s001.zip › botox.pdf]

Tarih: ....../....../......

TÜRK NÖROLOJİ DERNEĞİ

KAS İÇİ BOTULINUM TOKSİN UYGULAMASI BİLGİLENDİRME FORMU

Hasta Adı-Soyadı:

Protokol No:…………………

Sayın Hastamız,

Tıbbi durumunuz ve hastalığınızın tedavisi için size önerilen tıbbi tedavi ve tanıya yönelik tüm işlemler hakkında bilgi sahibi olmak en doğal hakkınızdır. Tıbbi tedavi ve cerrahi girişimlerin yararlarını ve olası olumsuzlukları öğrendikten sonra yapılacak işleme olur vermek ya da vermemek yine kendi kararınıza bağlıdır.

Bu açıklamanın amacı sizi korkutmak ya da endişelendirmek değil, sağlığınız ile ilgili konularda sizi daha bilinçli bir biçimde karara ortak etmektir. İsterseniz sağlığınız ile ilgili tüm bilgi ve belgeler size veya uygun göreceğiniz bir yakınınıza verilebilir. Yasal ve tıbbi zorunluluk taşıyan durumlar dışında bilgilendirmeyi istemeyebilirsiniz.

………………………………………… Hastalığınızın……………………………durumu nedeni ile kas içi **botulinum toksin** uygulaması gerekmektedir.

Botulinum Toksini nedir?

Bir bakterinin salgıladığı güçlü bir zehirden elde edilen ve sinirlerden gelen uyarıların kaslara geçmesini engelleyen bir maddedir, içine Botulinum Toksini enjekte edilmiş bir kasın kasılma gücü, ilacın dozu ile orantılı olarak azalır veya tamamen kaybolur. Bu özelliği nedeniyle Botulinum Toksini, kasları istemsiz ve kontrolsüz olarak kasan, distoni gibi hastalıklarda kas gevşetici bir ilaç olarak kullanılır, ayrıca estetik amaçlı kırışıklık giderme, aşırı terlemede ve son dönemde migren tedavisinde kullanılmaktadır. Botulinum toksininin etki süresi kişiden kişiye değişkenlik göstermekle birlikte, kişinin kasını kullanımına bağlı olarak 3-6 ay sürebilmektedir. Uygulama sayısı arttıkça uygulamalar arası genelde açılabilmektedir. Botulismus toksini tedavisi kişiye özgü olmalıdır. Uygulama noktaları ve dozu cinsiyete ve kasın kasılma gücüne göre ayarlanmalıdır.

Botulinum Toksini uygulaması

Öncelikle hastalığınızın niteliği ve şiddeti ile ilişkili olarak, hangi kaslarınızın ne kadar gevşetilmesi gerektiği belirlenir. Belirlenen bu kaslara gereken dozlarda Botulinum Toksini enjekte edilir. Enjeksiyon işlemi esas olarak diğer kas-içi ilaç veya aşı uygulamalarında olduğu gibidir; ilaç enjektöre çekilerek doğrudan kas içine verilir. Enjeksiyon için hedeflenen kasın yerini doğru olarak saptamak gerektiğinde işlem genellikle EMG (elektromyografi) yönteminin yardımı ile yapılır. EMG kaslardaki elektriksel aktiviteyi gösterir ve bu yolla kasın yerini saptar. Bu amaç için kullanılan EMG elektrodu aynı zamanda enjeksiyon iğnesidir; yani belli bir kas için EMG işlemi ve enjeksiyon işlemi aynı anda ve tek bir iğne batırışı ile gerçekleşir. Enjeksiyon işlemi diğer kas-içi ilaç enjeksiyonlarındakine benzer bir ağrıya neden olur; EMG kullanımının ağrıyı artırıcı bir etkisi yoktur.

Botulinum Toksinin etki süresi ve uygulama sıklığı

ilacın etkisi enjeksiyondan birkaç gün sonra başlar ve genellikle yaklaşık olarak 3 ay sürer. Bu süre sonunda sinirinden gelen uyarıları tekrar almaya başlayan kas yavaş yavaş eski gücüne kavuşur. Bu nedenle etkinin devamı için enjeksiyonun 3 ayda bir tekrarlanması gerekir. 3. ay sonunda ilacın etkisi henüz geçmemişse tekrar enjeksiyon için daha uzun bir süre de beklenebilir. 3 aydan daha kısa bir sürede enjeksiyon tekrarlanırsa ilaca karşı antikorlar

gelişmesi ve sonraki enjeksiyonlarda ilacın etkisinin kaybolması riski vardır. Bu nedenle, ilacın etkisi 3 aydan daha kısa bir sürede geçmiş olsa bile, yeniden bir enjeksiyon için en az 3 ay beklemek gerekir.

Botulinum Toksini başarı olasılığı

Uygulamadan ne kadar yarar göreceğiniz hastalığınızın niteliğine, ne kadar çok kası ilgilendirdiğine ve bu kastar arasındaki etkileşim miktarına bağlı olarak çok değişkendir. Hiçbir yararlı etki görülmeyebileceği gibi, %90'ın üzerinde bir iyileşme de beklenebilir.

Botulinum Toksini enjeksiyonunun yan etkileri ve uygulama ile ilgili uyarılar

Enjekte edilen kaslarda ve bu kaslara komşu diğer bazı kaslarda ortaya çıkan güçsüzlük ve buna bağlı geçici fonksiyon kayıpları en sık görülen yan etkilerdir. Bunlar arasında, enjekte edilen kasa bağlı olarak, yutma güçlüğü, kafanın öne düşmesi, göz kapağında ve kaşlarda düşme, çift görme, alerjik reaksiyonlar, el ve kol becerilerinin azalması görülebilir. Bunun dışında, enjeksiyon yerinde birkaç gün süren ağrılar görülebilir. Botulinum Toksinin kalıcı bir yan etkisi yoktur.

- Uygulamaya gelirken lütfen iyice dinlenmiş olarak geliniz.
- Uygulama öncesinde alkollü içecekler tüketmeyiniz.
- En az üç gün öncesinden gingobiloba, kan sulandırıcı ilaçlar, aspirin ve non-steroid antiinflamatuar (romatizmal) ilaçlar kesilmelidir.
- Uygulama sonrası Kas gevşetici ve antibiyotik kullanmanız gerektiğinde doktorunuza danışınız.
- Hamilelikten kaçının veya etkili bir gebelik önleme yöntemi kullanınız.
- Emzirmekten kaçının.

Botulinum Toksini tedavisi yerine farklı seçenekler

Botulinum Toksini enjeksiyonunun hastalığınızı "kökten" tedavi edici bir özelliği yoktur; sadece hastalığınızın sebep olduğu şikayetlerinizi geçici bir süre için -enjeksiyonlara devam edildiği sürece-düzeltir veya azaltır. Bu enjeksiyonları yaptırsanız da yaptırmasanız da hastalığınız kendi doğal seyri içinde devam eder. Hastalığınızın niteliğine bağlı olarak, Botulinum Toksini yaptırıyor olsanız bile, doktorunuzun önerdiği diğer bazı ilaçları da birlikte kullanmanız gerekebilir. Botulinum Toksini'den vazgeçmeniz durumunda, yine hastalığınızın niteliğine bağlı olarak, Botulinum Toksini'den beklenen yararı kısmen karşılayabilecek diğer bazı tedavi yöntemleri söz konusu olabilir.

Hastalığımın tanısı ile ilgili olarak yapılacak **Botulinum Toksin Enjeksiyonu uygulamasının**:

- Niteliği ve nasıl uygulanacağı,
- İncelemeden beklenen yararlar ve olası yan etkiler veya zararlar,
- Hangi durumlarda incelemenin yapılamayacağı,
- Bu incelemeyi yaptırmayı istemezsem sağlığımla ilgili olarak karşılaşabileceğim olumsuzluklar,
- Bu incelemenin yerini alabilecek diğer seçenekler konusunda ayrıntılı olarak bilgilendirildim.

Doktorun yetki, gözlem ve yönetimi altında, yukarıda açıklanan **Botulinum Toksin Enjeksiyonu uygulamasının** üzerimde/vekili olduğum hasta üzerinde gerçekleştirilmesine <u>izin veriyorum.</u>

Tarih	: ... /.... /....	Saat	: .. ..
Hasta Adı/Soyadı	:	İmza	:

Dosya No :

Hasta Yakını (*) Adı/Soyadı : İmza :

(*) Hastanın Yakını: Vasiyet altındakiler için vasi, reşit olmayanlar için anne-baba, bunların bulunmadığı durumlarda 1. derece kanuni mirasçılar.
