# Supplementary material for: Autonomous Assessment of Medical Consent Forms: Development and Preliminary Feasibility Demonstration of the Universal Health Communication Index (UHCI)
Source: Healthcare (Basel). 2026 Sep 10;14(18):2956. doi: 10.3390/healthcare14182956 (PMC13606284; doi:10.3390/healthcare14182956)
Supplement: Supplementary file 1 [file healthcare-14-02956-s001.zip › emg -tr.pdf]

Tarih: ....../....../......

TÜRK NÖROLOJİ DERNEĞİ
EMG (ELEKTROMYOGRAFİ) HASTA BİLGİLENDİRİLMİŞ ONAM FORMU

Hasta Adı-Soyadı:
Protokol No:..................

Sayın Hastamız,

Tıbbi durumunuz ve hastalığınızın tedavisi için size önerilen tıbbi tedavi ve tanıya yönelik tüm işlemler hakkında bilgi sahibi olmak en doğal hakkınızdır. Tıbbi tedavi ve cerrahi girişimlerin yararlarını ve olası olumsuzlukları öğrendikten sonra yapılacak işleme olur vermek ya da vermemek yine kendi kararınıza bağlıdır.

Bu açıklamanın amacı sizi korkutmak ya da endişelendirmek değil, sağlığınız ile ilgili konularda sizi daha bilinçli bir biçimde karara ortak etmektir. İsterseniz sağlığınız ile ilgili tüm bilgi ve belgeler size veya uygun göreceğiniz bir yakınınıza verilebilir. Yasal ve tıbbi zorunluluk taşıyan durumlar dışında bilgilendirmeyi istemeyebilirsiniz.

.... hastalığınızın...............................durumu nedeni ile EMG (elektromyografi) gerekmektedir.

EMG nedir ve nasıl yapılır?
EMG (elektromyografi} kasların, sinirlerin ve sinir köklerinin elektriksel özelliklerinin ölçülmesi yoluyla bu yapıların sağlık durumlarının değerlendirildiği bir muayene yöntemidir. EMG tetkiki iki kısımda yapılır:
1) Sinirlerin incelenmesi
2) Kasların incelenmesi

Tanıya göre her ikisinin veya yalnızca birinin yapılması gerekebilir. Sinir iletimlerinin ölçümünü doktor veya doktorun belirlediği şekilde bir EMG teknisyeni, kasların incelenmesi işlemi ise sadece doktor tarafından yapılır. İnceleme süresi yapılması düşünülen işlemin kapsamına göre 15 dakika ile 1.5 saat arasındadır.

1) Sinirlerin incelenmesi: Kol, bacak, veya diğer vücut kısımlarında seçilen bazı sinirler, deri üzerine yerleştirilen bir elektrot aracılığı ile elektrik akımı verilerek uyarılır. Elektrik akımı verildiği yerde hoşa gitmeyen fakat dayanılabilir bir his oluşturur. Verilen uyarı ile sinirde ortaya çıkan elektriksel aktiviteler çeşitli şekillerde ölçülür. Bir EMG incelemesi sırasında genellikle böyle 5-10 sinirden ölçüm yapılır.

2) Kasların incelenmesi: İğne şeklinde bir elektrot kol, bacak veya diğer vücut kısımlarında seçilen bir kas içine yerleştirilerek kastaki elektriksel aktiviteler incelenir. Kullanılan elektrot, enjeksiyon iğnelerine benzer boyut ve görünümdedir. Bu elektrotlar tek kullanımlıktır, her hastada yeni bir iğne kullanılır ve bu iğne inceleme bittikten sonra atılır (Tek-lif EMG'si gibi bazı tetkiklerde elektrotlar tek kullanımlık değildir, mikroptan arındırılarak yeniden kullanılır). Elektrot iğnesinin kas içine yerleştirilmesi sırasında duyulan ağrı kas içine ilaç enjeksiyonu yapılışı sırasında duyulan ağrıya benzer; fakat EMG'de herhangi bir madde enjeksiyonu yapılmadığı için ağrı daha az şiddetlidir. Her bir kasın incelenmesi birkaç dakika sürer. Bu süre içinde iğne kas içinde tutulur. Kas içinde değişik yerlerden kayıt alma amacıyla iğnenin yönü ve yerinin birkaç kez değiştirilmesi gerekebilir. Bu işlemler genellikle ağrısızdır veya katlanılabilir derecede hafif şiddette bir ağrı oluşturur. Bir EMG tetkikinde incelenmesi gereken kas sayısı düşünülen tanıya göre değişir; genellikle 1-10 arası sayıda kas incelenir.

EMG'nin, elektrikle uyarım ve iğne ile inceleme sırasında ortaya çıkan ağrı dışında beklenen bir yan etkisi ve bilinen kalıcı bir zararı yoktur, Ender olarak, incelenen kaslar içinde geçici bir kan birikimi oluşabilir. Çok daha ender olarak bazı derin gövde kaslarının incelenmesi sırasında, komşu doku ve organlarda geçici olumsuz etkiler ortaya çıkabilir.
İleri derecede kan pıhtılaşma bozukluğu olan veya kan pıhtılaşmasını engelleyen ilaç kullanan hastalarda zorunlu olmadıkça EMG incelemesi yapılmaz. Bunun dışında her yaş grubunda ve herkese yapılabilir; hastada kalp pili veya beyin pili bulunması EMG yapılmasına engel değildir.

Bire-bir EMG incelemesinin yerine geçen başka bir inceleme yoktur; EMG yaptırmayı istemediğiniz durumda, rahatsızlığınızın tanısına yardımcı olacak bazı bilgilerde eksiklik olabilir. Ancak, rahatsızlığın niteliğine bağlı olarak bazı durumlarda, EMG incelemesi ile elde edilmesi beklenen bilgi, kısmen diğer bazı inceleme yöntemleri ile de elde edilebilir.

Hastalığımın tanısı ile ilgili olarak yapılacak EMG incelemesi:

- Niteliği ve nasıl uygulanacağı,

- İncelemeden beklenen yararlar ve olası yan etkiler veya zararlar,
- Hangi durumlarda incelemenin yapılamayacağı,
- Bu incelemeyi yaptırmayı istemezsem sağlığımla ilgili olarak karşılaşabileceğim olumsuzluklar,
- Bu incelemenin yerini alabilecek diğer seçenekler konusunda ayrıntılı olarak bilgilendirildim.

Doktorun yetki, gözlem ve yönetimi altında, yukarıda açıklanan EMG incelemesinin üzerimde/vekili olduğum hasta üzerinde gerçekleştirilmesine izin veriyorum.

Tarih	: ... /.... /....	Saat	: .. ..
Hasta Adı/Soyadı	:	İmza	:
Dosya No	:		
Hasta Yakını (*) Adı/Soyadı	:	İmza	:

(*) Hastanın Yakını: Vasiyet altındakiler için vasi, reşit olmayanlar için anne-baba, bunların bulunmadığı durumlarda 1. derece kanuni mirasçılar.
