# Supplementary material for: Autonomous Assessment of Medical Consent Forms: Development and Preliminary Feasibility Demonstration of the Universal Health Communication Index (UHCI)
Source: Healthcare (Basel). 2026 Sep 10;14(18):2956. doi: 10.3390/healthcare14182956 (PMC13606284; doi:10.3390/healthcare14182956)
Supplement: Supplementary file 1 [file healthcare-14-02956-s001.zip › lp.pdf]

Tarih: ....../....../......

TÜRK NÖROLOJİ DERNEĞİ LOMBER PONKSİYON
(BEYİN OMURİLİK SIVISI İNCELEMESİ) BİLGİLENDİRME FORMU

Hasta Adı-Soyadı:
Protokol No:……………..

Sayın Hastamız,
Tıbbi durumunuz ve hastalığınızın tedavisi için size önerilen tıbbi tedavi ve tanıya yönelik tüm işlemler hakkında bilgi sahibi olmak en doğal hakkınızdır. Tıbbi tedavi ve cerrahi girişimlerin yararlarını ve olası olumsuzlukları öğrendikten sonra yapılacak işleme olur vermek ya da vermemek yine kendi kararınıza bağlıdır.

Bu açıklamanın amacı sizi korkutmak ya da endişelendirmek değil, sağlığınız ile ilgili konularda sizi daha bilinçli bir biçimde karara ortak etmektir. İsterseniz sağlığınız ile ilgili tüm bilgi ve belgeler size veya uygun göreceğiniz bir yakınınıza verilebilir. Yasal ve tıbbi zorunluluk taşıyan durumlar dışında bilgilendirmeyi istemeyebilirsiniz.

Hastalığınızın tanı/tedavisi için lomber ponksiyon gerekmektedir. Özel bir iğneyle bel kısmından girilip omuriliği kaplayan zarların geçilerek beyin-omurilik sıvısına ulaşılması işlemine lomber ponksiyon denir. Lomber ponksiyon en sık olarak beyin-omurilik sıvısı örneği elde etmek için yapılır. Beyin-omurilik sıvısının incelenmesi ile menenjit gibi bazı enfeksiyon hastalıklarının tanısı konulabilmektedir. Ayrıca, bazı kanser türlerinde beyine, omuriliğe ve bunları kaplayan zarlara yayılma olup olmadığını anlamak için, bazı kafa içi basınç artımı hastalıklarında multiple skleroz ve de sinir iltihabı durumlarında lomber ponksiyon yapılması gerekir. Lomber ponksiyon izlem ve tedavinin planlı bir bölümü olabilir Günümüzde bu girişimin yerini tutacak ve aynı amaçlara hizmet edecek alternatif yöntem yoktur. Girişim öncesinde doktorunuz sizinle ve bilinci yerindeyse hastanızla görüşerek işlem hakkmda bilgi verecektir, Hastanızın önemli tıbbi sorunları, kullandığı ilaçları, varsa kanama sorunları veya alerji durumlarını (ilaçlara, latekse, flastere vb) veya başka hastalıklarını girişimden önce doktorunuza mutlaka söylemelisiniz.

Lomber ponksiyon girişimi genellikle hasta yan yatırılarak (nadiren hasta oturtulup öne eğik pozisyon verilerek) bel bölgesindeki omurların arasındaki boşluktan yapılır. Hastaya pozisyon verildikten ve hareketsiz durması sağlandıktan sonra girişimin yapılacağı yerin üzerindeki cilt antiseptik solüsyonla silinerek üzeri delikli mikroptan arındırılmış örtü ile örtülür. Lomber ponksiyon yapılabilmesi için cilt ve cilt altının özel iğne ile delinmesi gerekir. İşlem sırasında iğnenin batırıldığı yerde ciltte ve cilt altı dokularda hasta iğne batmasına bağlı acı duyar. Bunu en aza indirmek için cilt ve cilt altına yerel uyuşturucu verilerek uyuşturulabilir. Yapılacak incelemeler için gereken en az miktarda bel sıvısı mikroptan arındırılmış tüplere alınacaktır. Bütün bu işlemler yaklaşık 10-15 dakika sürecektir. Lomber ponksiyon girişimi tamamlandıktan sonra girişimin yapıldığı yerde cilt üzerine mikroptan arındırılmış gazlı bez ile 2-3 dakika bası uygulanır, kanama ve sızma kontrolü yapılır. Sonra bu yer mikroptan arındırılmış gazlı bez ile kapatılır ve flaster yapıştırılır.

Lomber ponksiyon sonrasında hastanızın en az 30 dakika süreyle sırt üstü düz yatması gerekir. Eve veya odasına dönünce de izleyen 24 saatte mümkün olduğu kadar dinlenmesi gerekir. Girişim sonrasında 48 saat kadar süre ile spor gibi yorucu aktivitelerden kaçınılması gerekir.

İşleme ait Riskler:
- Lomber ponksiyon sonrası hasta baş ağrısı hissedebilir. Baş ağrısı hafif olabileceği gibi bazen şiddetli de olabilir. Baş ağrısı durumunda hastanın sırt üstü düz yatırılması rahatlatıcı olacaktır, Rahatlamazsa ağızdan parasetamol gibi ağrı kesici ilaç kullanılması gerekebilir. Böyle bir durumda doktorunuzla iletişime geçmeniz gerekir. Aspirin kanamaya eğilim yaratabileceğinden alınmamalıdır. Ayrıca bol sıvı alınması da baş ağrısını önler veya azaltır. Bazen de hastanın girişim yapılan yerde ağrısı olabilir, bu durum genellikle geçici olsa da ağrı kesici verilmesi gerekebilir. Yerel uyuşturma için kullanılan ilaca karşı nadiren alerjik tepki gelişebilir. Bu durumda doktorunuz size gerekli girişimi yapacaktır.

Seyrek olarak girişim yapılan yerden uzayan kanama veya beyin-omurilik sıvısı sızması veya girişim yerinde kızarıklık, sıcaklık artışı ve ağrı ile kendini gösteren enfeksiyon görülebilir. Bunlar uygun şekilde tedavi edilebilecek durumlardır. Hastaneden ayrıldıktan sonra böyle durumlar dikkatinizi çekerse hemen doktorunuzu arayıp durumu bildirmelisiniz.

Sorumlu doktor tarafından yapılacak olan bel sıvısı alma işlemi ve bu sırada doğacak olumsuzluklar (komplikasyonlar) ve işlem sırasında uymam gerekenler hakkında ayrıntılı bilgi aldım yukarıda yazılı olanları da okudum ve anladım. İşlem yapılmasını kabul etmediğim takdirde doğacak sonuçlarla ilgili sorumluluklar tarafıma aittir. Ortaya çıkabilecek olumsuzlukları bilerek bu işlemin yapılmasını kabul ediyorum/etmiyorum.

Hastalığımın tanısı ile ilgili olarak yapılacak Lomber Ponksiyon incelemesi:

- Niteliği ve nasıl uygulanacağı,
- İncelemeden beklenen yararlar ve olası yan etkiler veya zararlar,
- Hangi durumlarda incelemenin yapılamayacağı,
- Bu incelemeyi yaptırmayı istemezsem sağlığımla ilgili olarak karşılaşabileceğim olumsuzluklar,
- Bu incelemenin yerini alabilecek diğer seçenekler konusunda ayrıntılı olarak bilgilendirildim.

Doktorun yetki, gözlem ve yönetimi altında, yukarıda açıklanan Lomber Ponksiyon incelemesinin üzerimde/vekili olduğum hasta üzerinde gerçekleştirilmesine <u>izin veriyorum.</u>

Tarih	: ... /.... /....	Saat	: .. ..
Hasta Adı/Soyadı	:	İmza	:
Dosya No	:		
Hasta Yakını (*) Adı/Soyadı	:	İmza	:

(*) Hastanın Yakını: Vasiyet altındakiler için vasi, reşit olmayanlar için anne-baba, bunların bulunmadığı durumlarda 1. derece kanuni mirasçılar.
