# Supplementary material for: Autonomous Assessment of Medical Consent Forms: Development and Preliminary Feasibility Demonstration of the Universal Health Communication Index (UHCI)
Source: Healthcare (Basel). 2026 Sep 10;14(18):2956. doi: 10.3390/healthcare14182956 (PMC13606284; doi:10.3390/healthcare14182956)
Supplement: Supplementary file 1 [file healthcare-14-02956-s001.zip › pe-tr.pdf]

Tarih: ....../....../......

TÜRK NÖROLOJİ DERNEĞİ
PLAZMAFEREZ (PLAZMA DEĞİŞİM TEDAVİSİ) VE İMMUNABSORPSİYON YÖNTEMİ İLE TEDAVİ
HASTA BİLGİLENDİRİLMİŞ ONAM FORMU

Hasta Adı-Soyadı:
Protokol No:..................

Sayın Hastamız,
Tıbbi durumunuz ve hastalığınızın tedavisi için size önerilen tıbbi tedavi ve tanıya yönelik tüm işlemler hakkında bilgi sahibi olmak en doğal hakkınızdır. Tıbbi tedavi ve cerrahi girişimlerin yararlarını ve olası olumsuzlukları öğrendikten sonra yapılacak işleme olur vermek ya da vermemek yine kendi kararınıza bağlıdır.

Bu açıklamanın amacı sizi korkutmak ya da endişelendirmek değil, sağlığınız ile ilgili konularda sizi daha bilinçli bir biçimde karara ortak etmektir. İsterseniz sağlığınız ile ilgili tüm bilgi ve belgeler size veya uygun göreceğiniz bir yakınınıza verilebilir. Yasal ve tıbbi zorunluluk taşıyan durumlar dışında bilgilendirmeyi istemeyebilirsiniz.

Plazmaferez (Plazma Değişim Tedavisi) ve İmmunabsorpsiyon Yöntemi ile Tedavi: Bağışıklık sistemi ile ilgili nörolojik hastalıklarda kullanılmaktadır. MS'de plazmada özellikle sıvısal bağışıklık hücrelerinin ve ilgili diğer elemanların vücuttan uzaklaştırılmasını sağlar. Böylece hücresel bağışıklık sistemini de düzenleyici etkisi bulunmaktadır. İmmunabsorpsiyon yönteminde plazmaferezden farklı olarak plazmanın belli elemanları seçici olarak uzaklaştırılır.

MS'de ağır ataklarda hızlı düzelme sağlayabilir. Yine ağır MS ataklarında yüksek doz IVMP- pulse (pals) tedavi ve veya IVIG tedavisine yanıt alınmadığı durumlarda uygulanabilir.

Uygulama Şekli: Plazma değişim tedavisi için genellikle kasık bölgesinde veya köprücük kemiği altındaki büyük toplar damara (ven) kateter yerleştirilmesi gerekmektedir. Tedavi gün aşırı 5-7 kez uygulanır. Her uygulamada hesaplanan plazma volümün 1-1,5 katı kadar plazma değiştirilir.

Yan Etkiler: Kateter uygulaması tromboz (damarın kan pıhtısı ile tıkanması), enfeksiyon, pnömotoraks (akciğer zarının yırtılarak hava girmesi) riski taşır. Tansiyon düşüklüğü, genel enfeksiyon, alerjik reaksiyon, ani akciğer ödemi, kansızlık, kalp krizi, kullanılan pıhtı önleyicilere bağlı kanama, sıvı dengesinin bozulması, serum kalsiyum düşüklüğü, ölüm gibi ciddi komplikasyonlar görülebilir.
MS konusunda bana (veya hastama) önerilen atak tedavisi ile ilgili tüm bilgileri (ilaç etkileri, uygulanış yolu, uygulanış süresi, yapılacak rutin tetkikler, tedavi süresince uyacağım tıbbi kurallar, yan etkiler, riskler, yan etki ve risklerin azaltılmasına yönelik yapılacak işlemler vb) okudum ve doktorum tarafından bilgilendirildim. Tarafıma yapılan tüm açıklamaları ve bilgileri anlamış bulunmaktayım. Bana önerilen bu tedavinin uygulanması konusundaki kararı, belli bir düşünme süresi sonunda, maddi manevi baskı altında kalmaksızın, tamamen hür irademe dayanarak aldım. İstediğim takdirde yapılacak tedaviyi durdurma ve reddetme hakkımın saklı olduğu konusunda bilgilendirildim. İşlem yapılmasını kabul etmediğim takdirde doğacak sonuçlarla ilgili sorumluluklar tarafıma aittir. İmzalı bilgilendirilmiş onay formunun bir kopyası tarafıma verilecektir.

- ○ MS hastalığı için önerilen ……………………………… tedavisini kabul ediyorum.

- ○ MS hastalığı için önerilen ……………………………… tedavisini kabul etmiyorum.

Hasta veya Vekili:
Adres:

Tel No:

Tarih :

İmza:

Katılımcı ile Görüşen Hekim:
Adres:

Tel No:

Görüşme tanığı:
Adres:

Tel No:

Tarih:

İmza:
