# Supplementary material for: Autonomous Assessment of Medical Consent Forms: Development and Preliminary Feasibility Demonstration of the Universal Health Communication Index (UHCI)
Source: Healthcare (Basel). 2026 Sep 10;14(18):2956. doi: 10.3390/healthcare14182956 (PMC13606284; doi:10.3390/healthcare14182956)
Supplement: Supplementary file 1 [file healthcare-14-02956-s001.zip › Supplementary Material - UHCI Core Scoring Logic.pdf]

UHCI Core Scoring Logic and Threshold Implementations

1. Autonomy Module: Passive Voice Penalty (10% to 50% Threshold)

The algorithm calculates the ratio of passive sentences to total sentences. A ratio under 10% receives a perfect score, while exceeding 50% results in a score of zero. A linear penalty is applied in between.

```
def score_passive_voice(passive_count, total_sentences):
    ratio = passive_count / total_sentences

    # Heuristic Thresholds:
    # 0% - 10% -> 100 Points
    # > 50% -> 0 Points

    if ratio <= 0.10:
        score = 100.0
    elif ratio >= 0.50:
        score = 0.0
    else:
        # Calculate linear drop across the 0.10 to 0.50 range (0.40 diff)
        progress = (ratio - 0.10) / 0.40
        score = 100.0 * (1.0 - progress)

    return {"score": round(score, 1), "ratio": ratio}
```

2. Transparency Module: Medical Jargon Penalty (2% to 15% Threshold)

This function evaluates the density of unexplained medical jargon against the total word count. Ratios below 2% are optimal, whereas a density above 15% is severely penalized.

```
def score_medical_jargon(final_jargon_count, total_words):
    ratio = final_jargon_count / total_words

    # Heuristic Thresholds: < 2% -> 100, > 15% -> 0
    if ratio < 0.02:
        score = 100.0
    elif ratio > 0.15:
        score = 0.0
    else:
        # Calculate linear drop across the 0.02 to 0.15 range (0.13 diff)
        progress = (ratio - 0.02) / 0.13
        score = 100.0 * (1.0 - progress)
        score = max(0.0, score)

    return {"score": round(score, 1), "ratio": ratio}
```

3. System Burden Module: Legal/Defensive Terminology (1% to 8% Threshold)

Legal terminology ratio is strictly penalized to discourage defensive medicine artifacts. *(Note: While initial design considered a 5% max cap, empirical tuning set the absolute zero floor at 8% as reflected in the core code).*

```
def score_legal_jargon(legal_term_count, total_words):
    ratio = legal_term_count / total_words

    # Heuristic Thresholds:
    # 0% - 1% -> 100 Points
    # > 8% -> 0 Points

    if ratio <= 0.01:
        score = 100.0
    elif ratio >= 0.08:
        score = 0.0
    else:
        # Calculate linear drop across the 0.01 to 0.08 range (0.07 diff)
        progress = (ratio - 0.01) / 0.07
        score = 100.0 * (1.0 - progress)

    return {"score": round(score, 1), "ratio": ratio}
```

4. System Burden Module: Clinical Time Cost & Reading Speed (100 vs. 130 WPM)

Calculates the operational time cost by combining memory-recall reading speeds (130 WPM for English, 100 WPM for morphologically complex agglutinative Turkish) and estimated interaction times (2.5 minutes per AI-generated patient question).

```
def score_time_cost(word_count, lang, avg_questions):
    # Differentiate memory-recall reading speeds based on linguistic
morphology
    speed = 100.0 if lang == 'tr' else 130.0
    reading_time = word_count / speed

    # Add predicted clinical explanation time (2.5 mins per question)
    extra_time = avg_questions * 2.5
    total_minutes = reading_time + extra_time

    # Heuristic Thresholds:
    # 0 - 15 min: 100 Points
    # 15 - 30 min: Linear drop
    # > 30 min: 0 Points
    if total_minutes <= 15.0:
        score = 100.0
    elif total_minutes >= 30.0:
        score = 0.0
    else:
        progress = (total_minutes - 15.0) / 15.0
        score = 100.0 * (1.0 - progress)

    return {"score": round(score, 1), "total_minutes": total_minutes}
```

5. Medical Adequacy Module: Cosine Similarity Thresholds (0.45 and 0.60)

Uses SentenceTransformer and tensor operations to map semantic compliance. Employs a >0.45 similarity baseline for standard item matching, and a strict >0.60 threshold for critical gap and patient retention analysis.

```
import torch
from sentence_transformers import util


def calculate_semantic_retention(patient_retention_embeddings,
gold_standard_critical_embeddings):
    # Calculate Cosine Similarity Matrix between recalled concepts and
critical risks
    cos_sim = util.cos_sim(patient_retention_embeddings,
gold_standard_critical_embeddings)


    # Extract the highest semantic match score
    max_sim = torch.max(cos_sim).item()


    # Strict Heuristic Threshold for Clinical Safety
    if max_sim > 0.60:
        retention_score = 100.0
        status = "SUCCESS"
    else:
        retention_score = 0.0
        status = "FAILURE"


    return retention_score, status, max_sim
```
