# Supplementary material for: Autonomous Assessment of Medical Consent Forms: Development and Preliminary Feasibility Demonstration of the Universal Health Communication Index (UHCI)
Source: Healthcare (Basel). 2026 Sep 10;14(18):2956. doi: 10.3390/healthcare14182956 (PMC13606284; doi:10.3390/healthcare14182956)
Supplement: Supplementary file 1 [file healthcare-14-02956-s001.zip › tpa.pdf]

## İNTRAVENÖZ TROMBOLİTİK TEDAVİ GEREKLİ DURUMLARDA KULLANILAN BİLGİLENDİRİLMİŞ ONAM FORMU ÖRNEĞİ

Tarih: ........../........../20.......
**Hasta Adı ve Soyadı:** ........................................................................................................
**Protokol Numarası:** ..........................................................................................................
**Hasta Yakını ve Yakınlık derecesi:** ..........................................................................................

Hasta ..........................................................................................................’ın (olarak) beyin atardamar tıkanması nedeniyle felç geçirmekte olduğunu (olduğumu) öğrendim. Ayrıca tıkanma sonucu hayati tehlike olması yanı sıra ağır bir sakatlık ile yaşama riski (riskim) bulunduğunu da öğrendim.

Nöroloji uzmanı ........................................................................................ hastalık hakkında yeterli bir şekilde bana risk ve tedavi seçeneklerini anlattı. Açıklamaları ayrıntılı olarak dinledim, damar tıkanıklığının damar içinden tPA adlı ilacının verilmesi ile açılma ve bu suretle mevcut sakatlığın düzelme şansının olduğunu öğrendim. Ayrıca pıhtı çözücü ilacın hem beyinde hem de vücudun diğer kısımlarında hayati tehlike yaratabilecek şiddette kanamaya yol açabileceğini de öğrendim. Sorularım tatmin edici şekilde cevaplandırıldı. Damarın tedavi ile mutlaka açılma garantisi olmadığını da kavradım. İşlem için başka tıbbi merkez ve kişilerden görüş alabileceğim belirtildi. Tedavi etkinliğinin geçen zaman ile azaldığını da anladım.

Tedaviyi kabul etmediğim veya istediğim anda tamamlanmadan durdurulmasını talep ettiğimde buna uyulacağı ama bu durumda oluşabilecek bütün sonuçlardan sorumlu olacağımı biliyorum. Tedaviyi reddedersem hasta hekim ilişkisinde ve hastanedeki diğer işlem ve uygulamalarda olumlu veya olumsuz bir etki yaratmayacağını kavradım.

Tüm bu açıklamalardan sonra bana karar vermem için yeterince süre tanındı, sonuçta fayda ve zarar oranlarını hesaba katarak serbest iradem ile tPA tedavisinin uygulanmasını kendi rızam ile kabul ettim. İmzalı bilgilendirilmiş onam formunun bir nüshasının istediğim zaman bana verileceğini de öğrendim.

**Hasta veya yakını:**
**Adres:** .............................................................
**Telefon:** ..........................................................
**Tarih:** .........../................/20..........
**İmza:** ...............................................................

**Görüşme tanığı:**
**Adres:** .............................................................
**Telefon:** ..........................................................
**Tarih:** .........../................/20..........
**İmza:** ....................................................................

Türk Nöroloji Derneği Beyin Damar Hastalıkları Bilimsel Çalışma Grubu tarafından hazırlanmıştır.
